# Genetic and functional modulation by agonist MRS5698 and allosteric enhancer LUF6000 at the native A_3_ adenosine receptor in HL-60 cells

**DOI:** 10.1007/s11302-024-09992-z

**Published:** 2024-02-28

**Authors:** Zhan-Guo Gao, Weiping Chen, Ray R. Gao, Jonathan Li, Dilip K. Tosh, John A. Hanover, Kenneth A. Jacobson

**Affiliations:** 1 Molecular Recognition Section, Laboratory of Bioorganic Chemistry, NIDDK, National Institutes of Health, 9000, Rockville Pike, Bethesda, MD 20892 USA; 2 Genomics Core, NIDDK, National Institutes of Health, 9000, Rockville Pike, Bethesda, MD 20892 USA

**Keywords:** Adenosine receptor, HL-60 cells, Agonist, Positive allosteric modulator, Microarray

## Abstract

The A_3_ adenosine receptor (AR) is an important inflammatory and immunological target. However, the underlying mechanisms are not fully understood. Here, we report the gene regulation in HL-60 cells treated acutely with highly selective A_3_AR agonist MRS5698, positive allosteric modulator (PAM) LUF6000, or both. Both pro- and anti-inflammatory genes, such as IL-1a, IL-1β, and NFκBIZ, are significantly upregulated. During our observations, LUF6000 alone produced a lesser effect, while the MRS5698 + LUF6000 group demonstrated generally greater effects than MRS5698 alone, consistent with allosteric enhancement. The number of genes up- and down-regulated are similar. Pathway analysis highlighted the critical involvement of signaling molecules, including IL-6 and IL-17. Important upstream regulators include IL-1a, IL-1β, TNF-α, NF-κB, etc. PPAR, which modulates eicosanoid metabolism, was highly downregulated by the A_3_AR agonist. Considering previous pharmacological results and mathematical modeling, LUF6000’s small enhancement of genetic upregulation suggested that MRS5698 is a nearly full agonist, which we demonstrated in both cAMP and calcium assays. The smaller effect of LUF6000 on MRS5698 in comparison to its effect on Cl-IB-MECA was shown in both HL-60 cells endogenously expressing the human (h) A_3_AR and in recombinant hA_3_AR-expressing CHO cells, consistent with its HL-60 cell genetic regulation patterns. In summary, by using both selective agonists and PAM, we identified genes that are closely relevant to immunity and inflammation to be regulated by A_3_AR in differentiated HL-60 cells, a cell model of neutrophil function. In addition, we demonstrated the previously uncharacterized allosteric signaling-enhancing effect of LUF6000 in cells endogenously expressing the hA_3_AR.

**Supplementary Information:**

The online version contains supplementary material available at 10.1007/s11302-024-09992-z.

## Introduction

Adenosine is a modulator of numerous functions by acting on the four adenosine receptor (AR) subtypes [1–3]. One of the mechanisms of adenosine-promoted tissue protection and repair has been ascribed to its anti-inflammatory effect [4]. In this context, A_3_AR modulation has been suggested as a promising approach for treating inflammatory and ischemic conditions. Additionally, A_3_AR agonists have progressed in clinical trials [2, 5–7] despite A_3_AR having both pro- and anti-inflammatory effects in various cell types [8–12]. Furthermore, Cohen et al. [13] have demonstrated that the A_3_AR positive allosteric modulator (PAM) *N*-(3,4-dichloro-phenyl)-2-cyclohexyl-1*H*-imidazo[4,5-*c*]quinolin-4-amine (LUF6000) also induced anti-inflammatory effects in three experimental rodent models, by a mechanism related to decreased levels of the transcription factor nuclear factor-κ (NF-κB) p65 protein. Moreover, LUF6000 induced a slight stimulatory effect on the number of normal white blood cells and neutrophils.

The A_3_AR has generated tremendous interest in anti-inflammatory drug development [7]. Many early studies used Cl-IB-MECA and IB-MECA as selective A_3_AR agonists, currently used in clinical trials for liver conditions and psoriasis [5–7, 14]. However, in light of the only moderate A_3_AR-selectivity of Cl-IB-MECA and IB-MECA [14], a concentration of ≤10 nM is often advisable for A_3_AR-specific analysis. Alternatively, A_1_ and A_2A_AR antagonists can be combined with Cl-IB-MECA to isolate A_3_AR’s role. For example, Wagner et al. [9] pre-administered A_2A_AR antagonist ZM241385 (1 mg/kg body weight) before Cl-IB-MECA treatment to reveal potential unspecific effects of Cl-IB-MECA on the A_2A_AR. With AR subtype selectivity in mind, Lillo et al. [15] performed RNAseq using striatal primary microglial cultures activated and treated with Cl-IB-MECA (200 nM) and/or A_2A_AR antagonist SCH58261 (200 nM). These results ultimately demonstrated that the chronic (24 h) A_3_AR agonist treatment regulated more genes negatively than positively. The associated gene ontology enrichment analysis showed the regulation of genes that participate in immune-related events. Analysis of protein-protein interactions showed that Smad3 and Sp1 genes are regulated by Cl-IB-MECA [15]. Under the conditions of cell activation with an agonist treatment regimen, Cl-IB-MECA tended not to favor gene expression related to neuroprotective microglia. Microarray or RNA-Seq has been used to explore anti- or pro-inflammatory functions of AR modulation in several cell types, including macrophages and microglia [15–17]. However, to our knowledge, the role of A_3_AR in neutrophils has not been explored using genetic approaches such as microarray or RNAseq.

Neutrophils are immune cells that are an important part of the host defense system against infection and tissue injury [18]. In response to these challenges, neutrophils migrate from peripheral blood toward the site of infection through the endothelial cell layer and into tissues [19]. Thus, the pharmacological modulation of neutrophil functions could enhance neutrophil capability against infections and protect tissues during inflammation. In the present study, we studied differentiated HL-60 cells, a commonly used cell line to model neutrophil functions such as cytokine release and migration [20–23].

The human (h) A_3_AR endogenously expressed in HL-60 cells has been characterized functionally and using radioligand binding [24, 25]. It was suggested that A_3_AR expression in HL-60 cells is like that in neutrophils, thus providing a practical model for investigating A_3_AR’s role. It is noted that A_2A_AR is also expressed in HL-60 cells [25]. Through studying both human neutrophils and HL-60 cells, Chen et al. [22] suggested that A_3_AR appears as the crucial AR for regulating neutrophil chemotaxis by controlling cell migration. Both A_3_AR agonists [26] and A_3_AR PAMs [13] are of interest as anti-inflammatory agents.

In the present study, we investigated gene regulation following the acute treatment of HL-60 cells with the recently available and highly selective A_3_AR agonist MRS5698, which we reported to be > 3000-fold receptor subtype selective at both human and rat A_3_AR [27], and the heterocyclic A_3_AR-selective PAM LUF6000. In addition to genetic modulation by the exogenous agonist, we also studied the allosteric enhancement by LUF6000 of the endogenously expressed HL-60 cell A_3_AR, which lacks previous exploration. It remains to be seen if chronic administration of MRS5698 and LUF6000 will be similar to or different than the chronic effects of Cl-IB-MECA, as reported by Lillo et al. [15].

## Materials and methods

### Materials

The MicroArray chips were purchased from Affymax (New York, NY, USA). Nonselective AR agonist adenosine-5′-*N*-ethyluronamide (NECA) and other reagents were from Sigma (St. Louis, MO, USA). (1*S*,2*R*,3*S*,4*R*,5*S*)-4-(6-((3-Chlorobenzyl)amino)-2-((3,4-difluorophenyl)ethynyl)-9*H*-purin-9-yl)-2,3-dihydroxy-*N*-methylbicyclo[3.1.0]hexane-1-carboxamide (MRS5698) was prepared as reported [27]. Our HL-60 cells were from ATCC (Manassas, VA, USA). An AlphaScreen cAMP kit was purchased from PerkinElmer (Waltham, MA). Other CHO cell lines stably expressing the hA_3_AR were made at the Laboratory of Bioorganic Chemistry, NIDDK (Bethesda, MD, USA). All other reagents were from standard commercial sources and of analytical grade. *N*-(3,4-Dichloro-phenyl)-2-cyclohexyl-1 H-imidazo[4,5-c]quinolin-4-amine (LUF6000) was synthesized at Leiden/Amsterdam Center for Drug Research (Leiden, The Netherlands) as reported [28]. Calcium assay kits were from Molecular Devices (Sunnyvale, CA, USA).

### Cell culture, drug treatment, and cell differentiation

CHO-A_3_ cells were grown in DMEM/F12 medium with 10% fetal bovine serum and 1% penicillin-streptomycin. HL-60 cells were cultured in RPMI-1640 medium (ATCC, Manassas, VA, USA) supplemented with 10% fetal bovine serum and 1% penicillin-streptomycin. Differentiation of HL-60 cells was accomplished by incubating cells with 1.3% DMSO for seven days before experiments. Our microarray analysis utilized four experimental groups, including a control group, MRS5698 group (1 µM, 20 min treatment), LUF6000 group (3 µM, 20 min treatment), MRS5698 + LUF6000 group (LUF6000 treatment for 20 min, followed by MRS5698 for 20 min). DMSO (5 mM) was used to dissolve the drugs before dilution in aqueous medium.

### RNA extraction and real-time PCR

Total RNA was extracted from 10^7^ differentiated HL-60 cells using a RNeasy kit (Qiagen, Redwood City, CA, USA) and was reversed-transcribed with the High-Capacity cDNA Reverse Transcription Kit (Applied Biosystems, Foster City, CA, USA) according to the manufacturer’s protocol. The cDNA was then amplified with gene-specific primers for each of the four AR subtypes, two cytokines IL-1± and IL-1², and GAPDH on a 7900HT Fast Real-Time PCR System (Applied Biosystems, Foster City, CA, USA) according to the manufacturer’s protocol. The TaqMan gene expression assays were from Applied Biosystems (Foster City, CA, USA). Values were normalized to GAPDH and then expressed as relative expression levels. The ΔΔCt method was used to conduct quantitative analysis of data.

### Intracellular calcium mobilization

The measurement of calcium mobilization was essentially described previously [29, 30]. Briefly, CHO cells expressing the recombinant hA_3_AR were grown in a set of 96-well black-wall, clear-bottom plates overnight before the day of the experiment and maintained at 37 °C/5% CO_2_. Then, we added 100 µl of dye (Calcium 6) to each well. Afterward, the plates were subsequently maintained at room temperature for 60 min. Finally, 50 µl of each compound or a control agonist was added into the respective assay plate wells during the determination of intracellular Ca^2+^ using a FLIPR (Molecular Devices, San Jose, CA, USA). We utilized a calcium assay kit as directed without washing cells and with probenecid added to the loading dye at a final concentration of 2.5 mM to increase dye retention. The compound plate was prepared using dilutions of various compounds in Hank’s Buffer (pH 7.4). Samples were run in duplicate at room temperature. Cell fluorescence (excitation = 485 nm; emission = 525 nm) was monitored following compound exposure. Increases in intracellular Ca^2+^ are reported as the maximum fluorescence value after exposure minus the basal fluorescence value before exposure.

### Measurement of cyclic AMP levels

CHO-A_3_ cells were cultured in DMEM/F12 (1:1) medium containing 10% fetal bovine serum, 100 units/ml penicillin, 100 µg/ml streptomycin, and 2 µmol/ml glutamine. For the assay of 3′,5′-cyclic adenosine monophosphate (cAMP), cells were plated in 96-well plates in 100 µl of medium overnight. An AlphaScreen cAMP kit was used for the measurement of cAMP levels as described previously [29, 30]. HL-60 cells 4 × 10^4^ cells/well were suspended in 80 µl Hank’s buffer containing 20 mM HEPES. Where applicable, the A_3_AR allosteric enhancer LUF6000 (3 µM) was added, and the incubation was continued for 20 min in the presence of phosphodiesterase inhibitor rolipram (10 µM), followed by the addition of A_3_AR agonist MRS5698 plus 10 µM forskolin to the mixture and incubation for 10 min. The reaction was terminated by centrifugation at 250 *g* for 5 min at 4°C upon the addition of 100 µL cold 0.3% Tween-20 to each well. Cells were then shaken at room temperature for 10 min. Finally, the reaction was terminated by supernatant removal, and the cells were lysed with the addition of 50 µl of lysis buffer (0.3% Tween-20). For the determination of cAMP production, an AlphaScreen cAMP kit was used according to the manufacturer’s instructions (PerkinElmer, Waltham, MA).

### Statistical and data analyses

The microarray RNA normalized data sets were loaded into the commercial bioinformatics software Partek Flow and were aligned with human genome Hg38 alongside the aligner BWA. The gene ANOVA result lists three treatment groups (Group 1, MRS5698, final concentration (1 µM); Group 2 LUF6000, final concentration (3 µM); Group 3, MRS5698 + LUF6000) in comparison to a corresponding control group, which were filtered by both *P* value of ≤0.05 and an absolute value of fold change ≥1.8. These filtered gene lists were then both used to create a Heat map using Partek Genomic Suite (Chesterfield, MO, USA) and a gene pathway analysis using the commercial software IPA (Qiagen, Redwood City, CA, USA). Functional parameters were calculated using Prism 10.0.0 software (GraphPAD, San Diego, CA, USA). Data was expressed as mean ± standard error. A Student’s *t-test* (between two conditions) or a One-Way Analysis of Variance (ANOVA) followed by Bonferroni’s multiple comparison tests (between multiple conditions) was used to compare statistically significant differences. Differences yielding *P* values < 0.05 are considered statistically significant.

## Results

### Genetic modulation by A_3_AR selective agonist MRS5698 and allosteric enhancer LUF6000

Table 1 shows the top ten genes upregulated (the complete list of genes is listed in Table S1). Following the treatment of MRS5698 (1 µM) for 20 min, many inflammation-associated genes were found upregulated 3-fold or more. For example, IL1A and IL1B, which are known for their inflammatory functions, are among the most highly upregulated genes. Interestingly, the anti-inflammatory IL-1RN, which reduces the inflammatory effects of cytokines IL-1α and IL-1β and aids the preservation of cell functions, was also highly upregulated. Both NFKBIA and NFKBIZ were also upregulated. NFKBIZ is a regulator of NFKB that inhibits NF-κB activity without affecting its nuclear translocation upon stimulation [31]. NFκB1A is an NF-κB response suppressor that attenuates inflammation, aging, and cancer [32]. TNFAIP3, the tumor necrosis factor α-induced protein 3, has been reported as a key molecule controlling NF-κB activation and has been linked to the development of multiple inflammation-related conditions in humans [33]. CCL4 has been shown to enhance preosteoclast migration alongside its receptor [34]. Additionally, CCR5 downregulation by RANKL promotes osteoclastogenesis [34]. Neutrophil-produced CCL4L2 was negatively related to inhaled corticosteroid (ICS) responsiveness in asthmatics and has been suggested as a potential therapeutic target for ICS-refractory asthma [35]. CCL3L3 has been suggested to be involved in susceptibility to systemic lupus erythematosus, an autoimmune disease [36]. Of the two most upregulated genes IL1A and IL1B, we confirmed the result using quantitative real-time PCR analysis. MRS5698 upregulated IL-1α and IL-1β mRNA expression by 19.1- and 16.4-fold, respectively, which is consistent with the data from microarray analysis.

**Table 1:** Fold-change of selected genes that were upregulated by treatment with A_3_AR agonist (MRS5698) alone or with PAM (LUF6000) or both^a^

Gene Symbol	Fold-Change (MRS5698 vs. Con)	Fold-Change (LUF6000 vs. Con)	Fold-Change (MRS5698 + LUF6000 vs. Con)
TNFAIP3	5.1	2.0	7.5
IL1B	43.0	7.6	52.5
NFKBIA	3.4	2.0	3.8
CCL4	5.9	2.3	6.1
CCL4L2	7.7	2.5	9.5
IL1A	56.7	5.7	98.6
CCL3L3	6.2	2.2	6.7
IL1RN	7.8	2.0	11.4
IER3AS1	8.6	2.6	11.0
NFKBIZ	15.7	4.9	20.4

^a^HL-60 cells were cultured in RPMI-1640 medium (ATCC, Manassas, VA) supplemented with 10% fetal bovine serum and 1% penicillin-streptomycin. Differentiation of HL-60 cells was accomplished by incubating cells with 1.3% DMSO for seven days before experiments. Four groups were divided for microarray analysis. Control group; MRS5698 group (1 µM, 20 min treatment); LUF6000 group (3 µM, 20 min treatment); MRS5698 + LUF6000 group (LUF6000 treatment for 20 min followed by MRS5698 for 20 min). Total RNA was extracted from 10^7^ differentiated HL-60 cells using a RNeasy kit (Qiagen, Redwood City, CA, USA). The microarray RMA normalized data sets were loaded into commercial bioinformatics software Partek Flow and were aligned with human genome Hg38 with aligner BWA. The complete list of genes regulated by A_3_AR activation is shown in Table S1

The changes in the LUF6000 (3 µM, 20 min) group were modest in comparison to the MRS5698 group (Table 1). The MRS5698 + LUF6000 group (LUF6000 was added 20 min before MRS5698 and incubated for an additional 20 min) showed overall larger upregulation compared with the MRS5698 group. This pattern of gene expression by LUF6000 is consistent with its functional enhancement [30]. The complete list of genes with significant upregulation or downregulation is listed in Table S1 (Supporting Information).

**Fig. 1:**
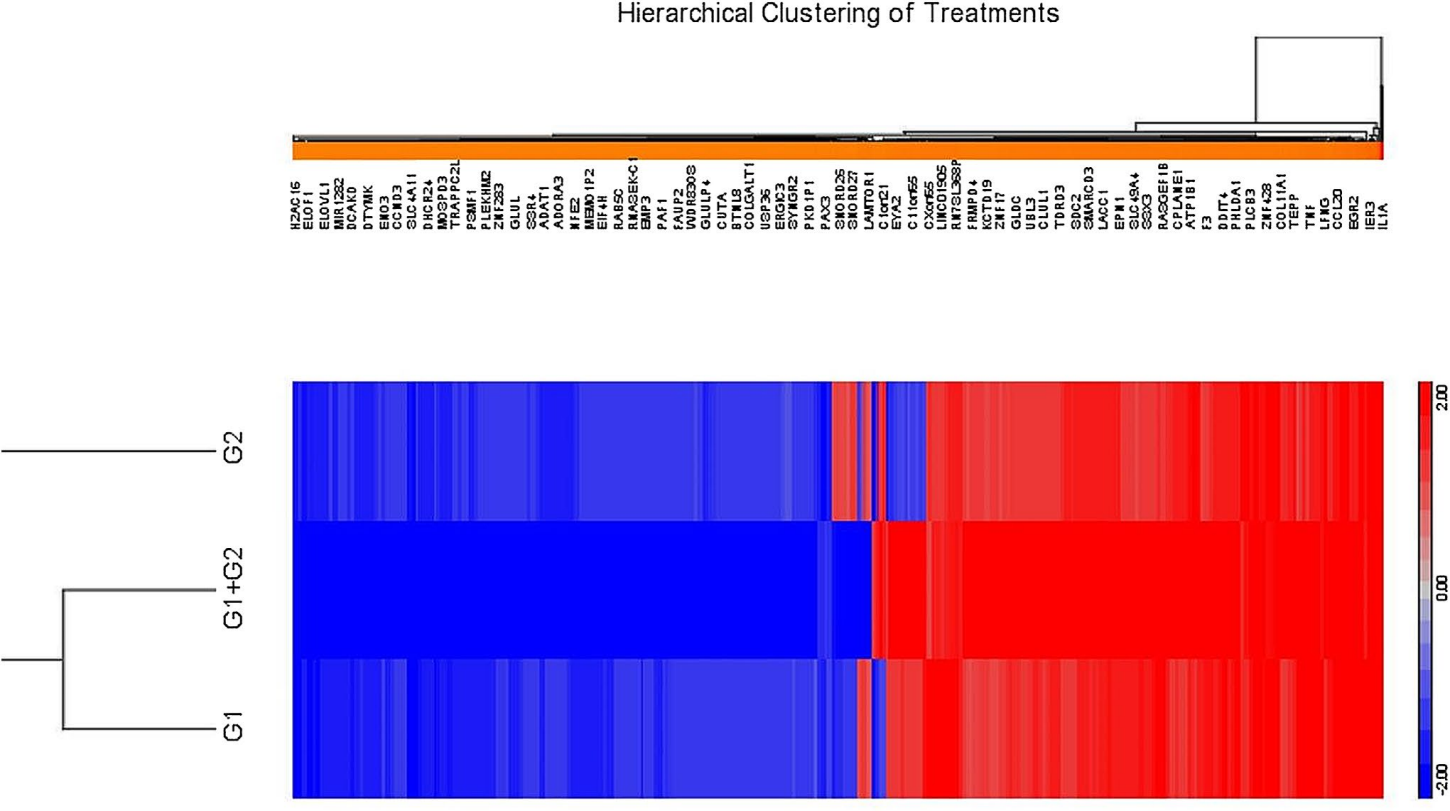
List of genes were upregulated and downregulated in differentiated HL-60 cells comparing all three groups following acute A_3_AR modulator treatment: MRS5698 (G1), LUF6000 (G2), and MRS5698 + LUF6000 (G3)

Notably, a similar number of genes were upregulated and downregulated upon MRS5698 treatment (20 min) (Fig. 1). These results contrast with a recent report by Lillo et al. [15], reporting that chronic treatment (24 h) of activated mouse microglia with another A_3_AR agonist Cl-IB-MECA resulted in mostly downregulated genes. The upregulation by LUF6000 and MRS5698 + LUF6000 is generally consistent with that in the MRS5698 alone group.

It is also interesting to note that the heatmap shows that LUF6000 enhanced both the down- and upregulation of genes regulated by the selective A_3_AR agonist MRS5698 (Fig. 1). Furthermore, even in the absence of an exogenous agonist, the allosteric enhancer LUF6000 alone shows an effect in the regulation of many genes in the same direction as the agonist MRS5698, which is consistent with an early report that another A_3_AR PAM, LUF6096, induced anti-ischemic cardioprotective effect in the absence of an exogenous agonist [37].

**Fig. 2:**
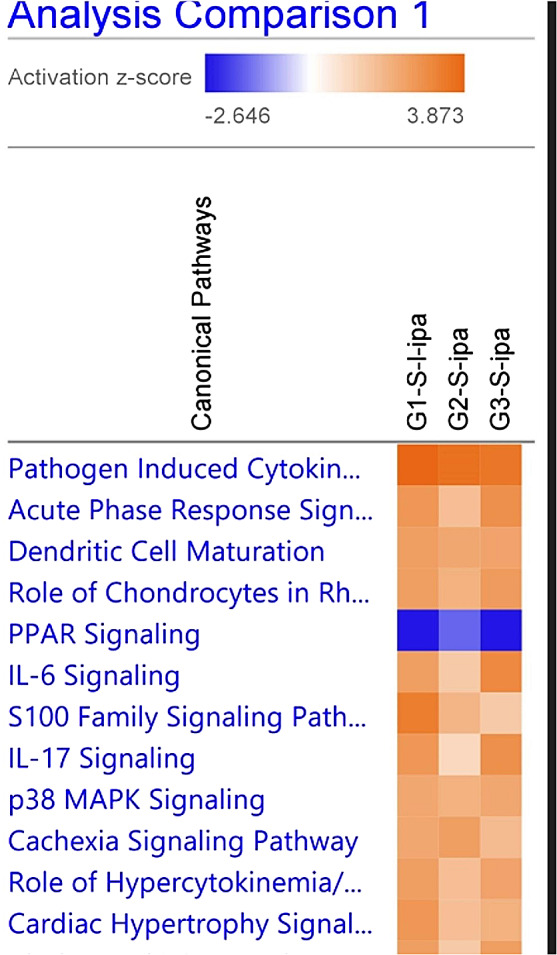
Pathway analysis for genes were upregulated and downregulated in differentiated HL-60 cells upon acute treatment with A_3_AR modulators: MRS5698 (G1-S-1-ipa), LUF6000 (G2-S-ipa), and MRS5698 + LUF6000 (G3-S-ipa)

Pathway analysis (Fig. 2) highlighted the critical involvement of important signaling pathways downstream of the A_3_AR, including IL-6, IL-17, and P38 MAPK signaling, as evidenced by all treatment groups: MRS5698 (G1-S-1-ipa), LUF6000 (G2-S-ipa), MRS5698 + LUF6000 (G3-S-ipa). The complete list of pathways from IPA analysis is included in the Supplementary Information (Figure S1). Canonical peroxisome proliferator-activated receptor (PPAR) and adrenergic receptor signaling pathways are strongly downregulated in both agonist groups and somewhat downregulated even in the LUF6000 group. Retinoic acid receptor (RAR) activation and liver X receptor-retinoid X receptor (LXR/RXR) gene expression is also downregulated in both agonist groups.

**Fig. 3:**
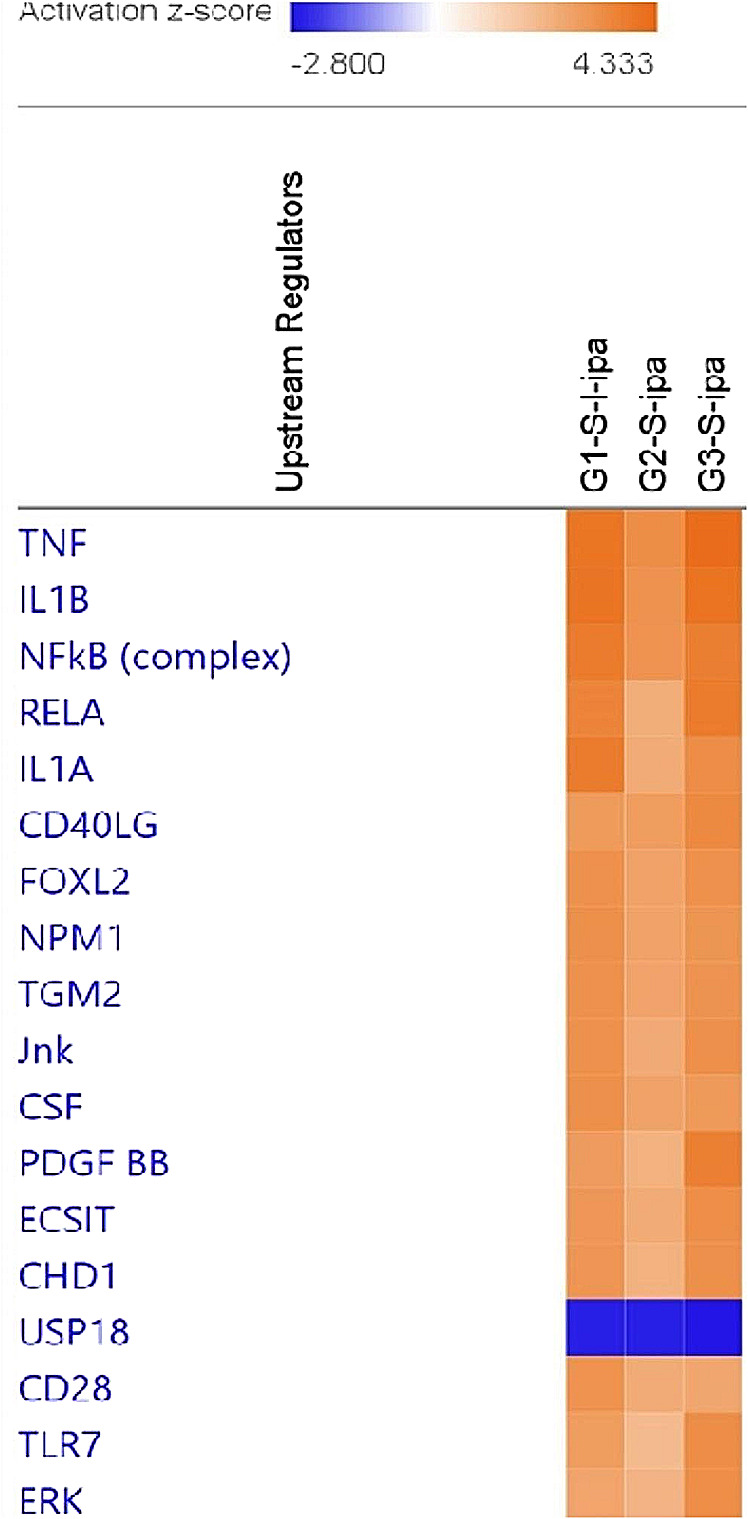
UPS analysis of regulators from all three HL-60 cell treatment groups, MRS5698 (G1-S-1-ipa), LUF6000 (G2-S-ipa), and MRS5698 + LUF6000 (G3-S-ipa), showing only the top genes. The complete list is found in Figure S2 (Supporting Information)

Upstream (UPS) analysis of regulators from all three groups MRS5698 (G1-S-1-ipa), LUF6000 (G2-S-ipa), and MRS5698 + LUF6000 (G3-S-ipa) identified important regulatory molecules including IL-1α, IL-1β, TNF-α, NFκB, JNK and ERK (Fig. 3). The complete list of upstream regulators from IPA analysis is included in the Supplementary Information (Figure S2). Analysis of known network interactions and regulated genes reveals IL-1β, IL-17, and TNF as important elements connecting A_3_AR signaling and function in vivo.

**Fig. 4:**
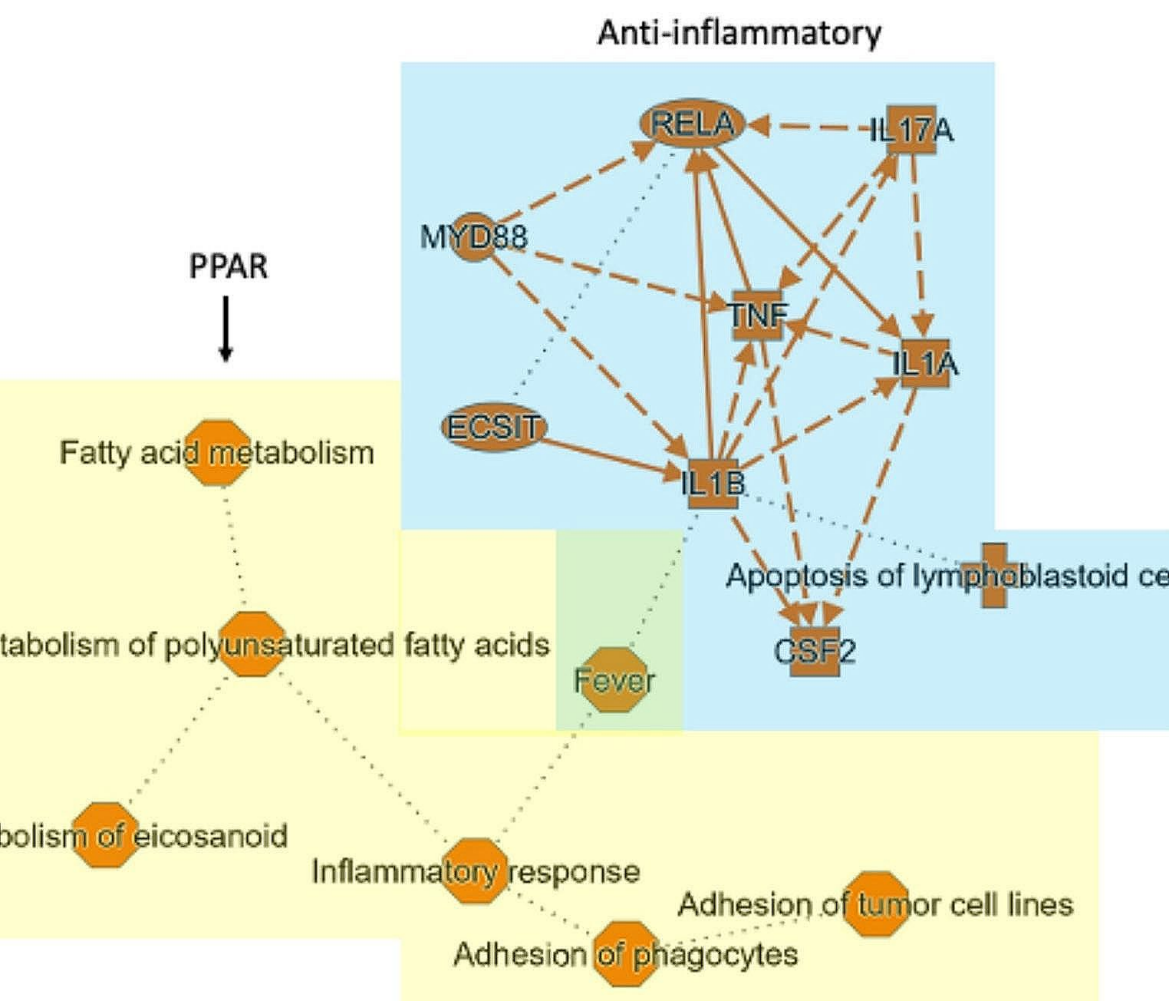
IPA analysis summary of the MRS5698 + LUF6000 group. Two pathway nodes are highlighted: PPAR (yellow) and anti-inflammatory (blue)

The IPA analysis summary of the MRS5698 + LUF6000 group is shown in Fig. 4. Two partially overlapping pathway nodes are evident: downstream of PPAR, which is highly downregulated by an A_3_AR agonist, and an anti-inflammatory node. The modulation of eicosanoid/arachidonic acid pathways downstream of PPAR would demonstrate mixed effects on inflammation and metabolism.

### Selective A_3_AR agonist MS5698: functional characterization at four AR subtypes

**Fig. 5:**
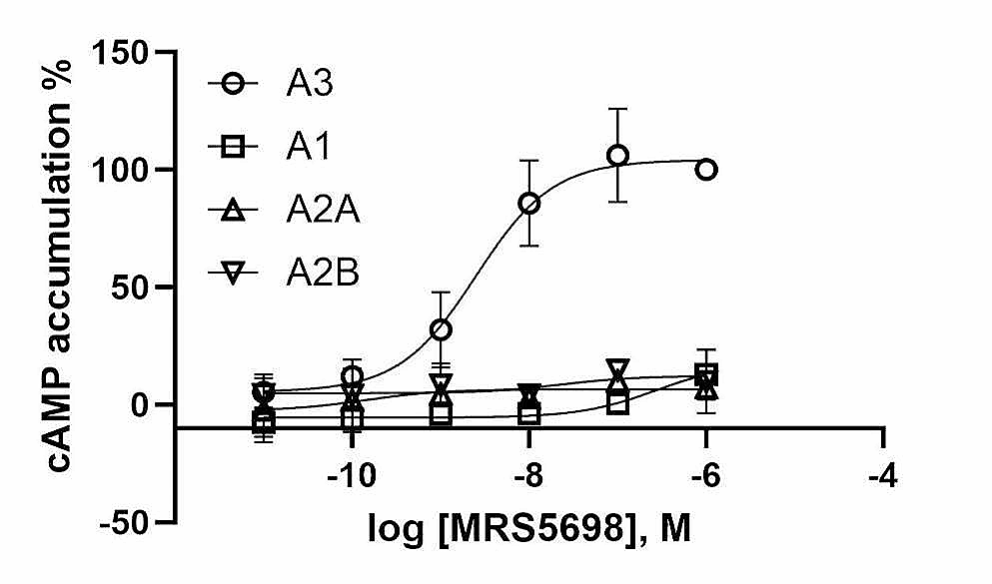
MRS5698 activates the hA_3_AR overexpressed in CHO cells, to inhibit forskolin-stimulated cAMP accumulation (EC_50_ = 2.52 nM)

The moderately selective A_3_AR agonists IB-MECA and Cl-IB-MECA have been used in many previous pharmacological studies. As mentioned above, at higher concentrations both agonists may activate other AR subtypes. Thus, we chose a more highly selective A_3_AR agonist in the present study. MRS5698 has been shown in radioligand binding assays to be a selective agonist for both human and mouse A_3_AR compared to A_1_AR and A_2A_AR [27]. However, its activation profile at four ARs has not been compared. To ensure that the effect of MRS5698 used in the genetic regulation is solely via the A_3_AR, we determined the effect of MRS5698 on cAMP accumulation in CHO cells expressing the recombinant human A_3_AR. Figure 5 shows that MRS5698 is potent in inhibiting forskolin-stimulated cAMP accumulation (EC_50_ = 2.52 nM) in hA_3_AR-expressing cells, and the nucleoside was largely inactive as an agonist at hA_1_, A_2A_, and A_2B_ARs at concentrations up to 1 µM (Fig. 5). Thus, we selected a concentration of 1 µM to treat HL-60 cells.

### Enhancement of A_3_AR function in HL-60 cells by LUF6000

**Fig. 6:**
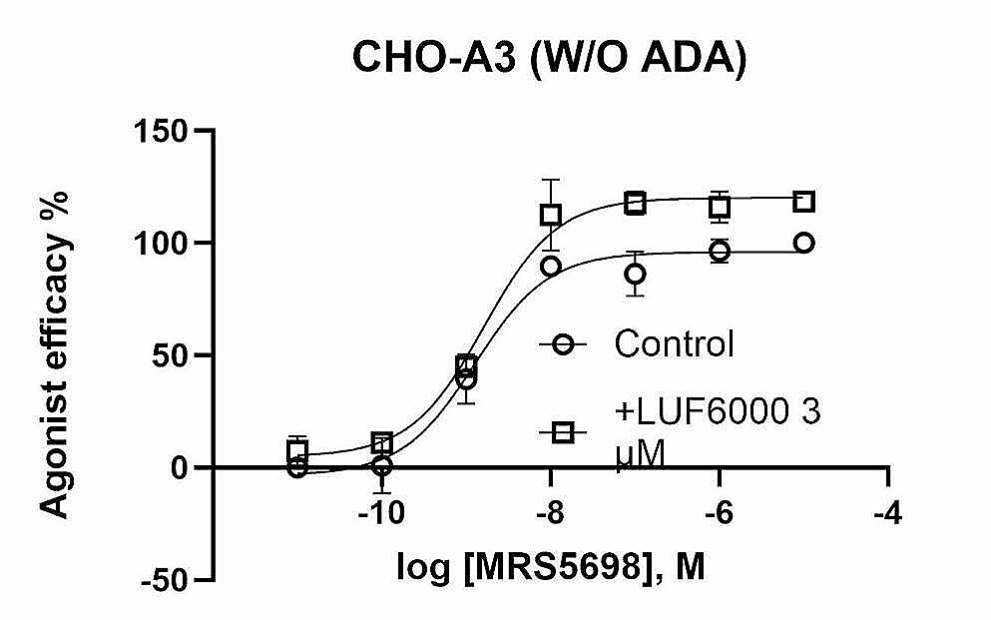
LUF6000 enhances the maximal activation (19% E_max_ enhancement) by MRS5698 of the hA_3_AR overexpressed in CHO cells, to further inhibit forskolin-stimulated cAMP accumulation. EC_50_ of control, 1.27 nM; +LUF6000, 1.61 nM

LUF6000 at a concentration of 3 µM has demonstrated a substantial enhancement of the A_3_AR agonist E_max_ without affecting agonist potency [30]. It has also been shown that LUF6000 is more efficacious in enhancing the E_max_ of partial agonists than full agonists [30]. At a concentration of 3 µM, LUF6000 enhanced the E_max_ of Cl-IB-MECA by 41% in an assay of cAMP accumulation [30], while it enhanced the E_max_ of the full agonist NECA by less than 20%. Here we show that it enhanced the E_max_ of MRS5698 by 19%, which suggests that MRS5698 is probably a nearly full agonist (Fig. 6).

**Fig. 7:**
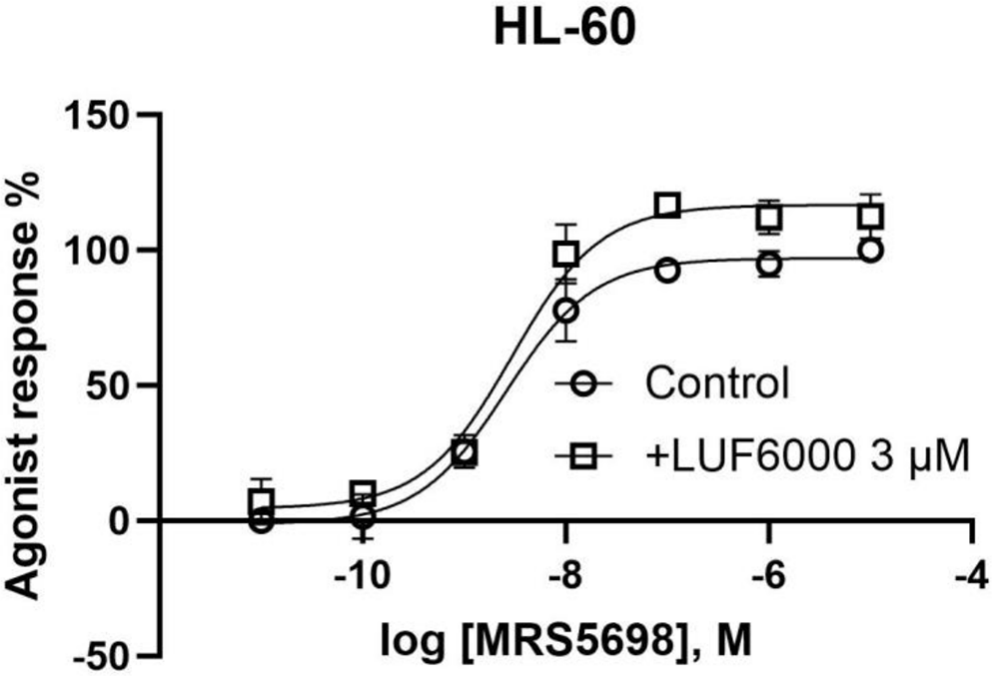
LUF6000 enhances the maximal activation (20% E_max_ enhancement) by MRS5698 of the hA_3_AR endogenously expressed in differentiated HL-60 cells, to further inhibit forskolin-stimulated cAMP accumulation. EC_50_ of control, 2.60 nM; +LUF6000, 2.95 nM

We further tested the function of MRS5698 and its enhancement by LUF6000 in differentiated HL-60 cells. Similar to CHO cells expressing the recombinant hA_3_AR, HL-60 cells endogenously expressing the hA_3_AR LUF6000 also enhanced the agonist efficacy of MRS5698 by ~ 20% (Fig. 7).

### MRS5698-induced intracellular calcium mobilization

**Fig. 8:**
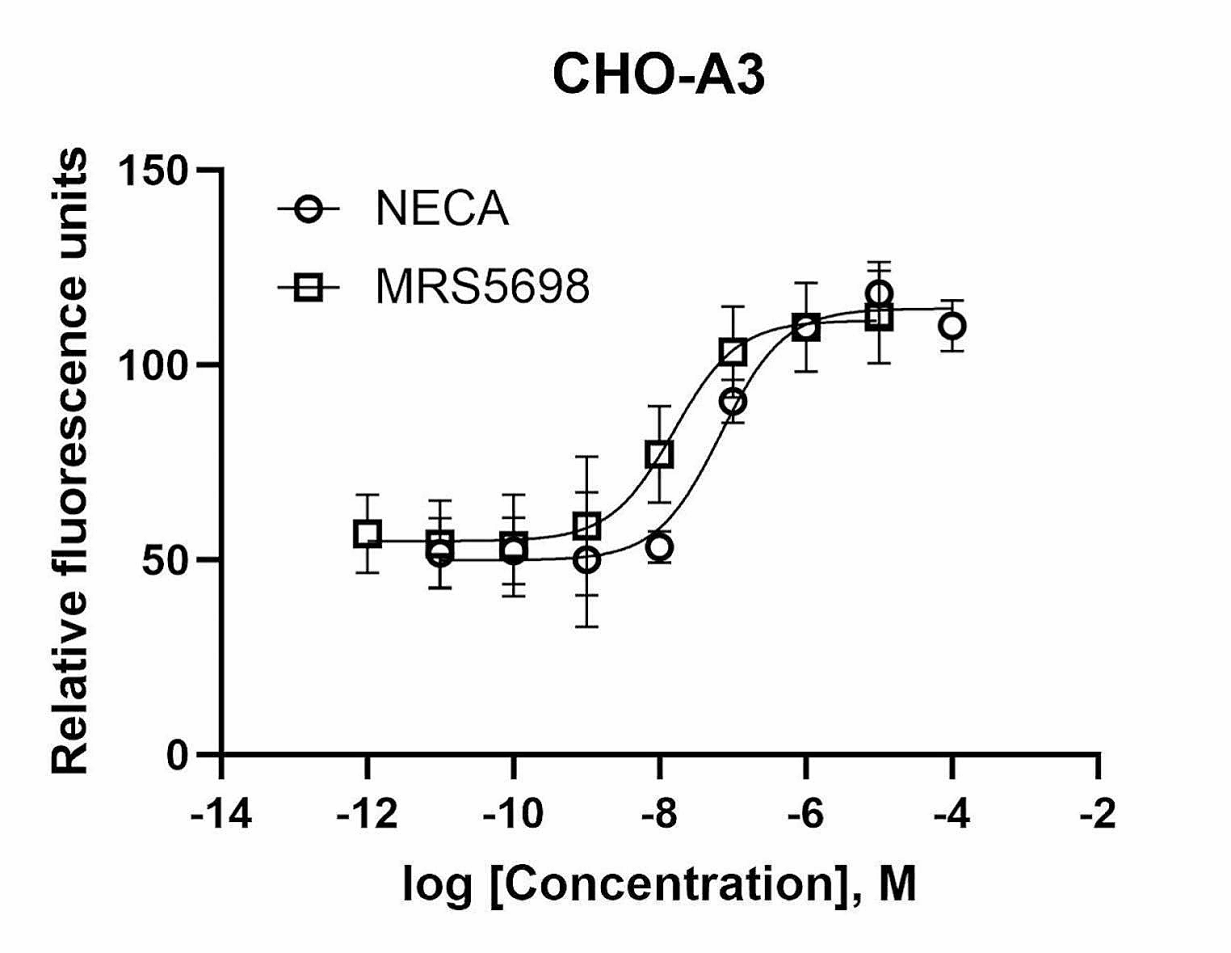
Calcium mobilization assay in CHO-A3 cells, showing that MRS5698 and NECA produce equi-efficacious hA_3_AR agonism, but MRS5698 is more potent. EC_50_ values: NECA, 69.2 ± 17.2 nM; MRS5698, 17.3 ± 4.7 nM

It has not been previously characterized whether MRS5698 is a full agonist or a partial agonist. The relatively small enhancement by LUF6000 on MRS5698-induced gene upregulation alongside the smaller effect of LUF6000 on the E_max_ of MRS5698 in the cAMP assay in comparison to the relatively larger effect of LUF6000 on another known partial agonist Cl-IB-MECA [30] suggests that MRS5698 is likely a nearly full A_3_AR agonist. Thus, in the present study, we compared MRS5698 with a known full A_3_AR agonist NECA [30] in a calcium mobilization assay in CHO-A3 cells. Figure 8 shows that MRS5698 is as efficacious as NECA as an A_3_AR agonist but 4.0-fold more potent.

### Gene expression comparison of the four AR subtypes in differentiated HL-60 cells

**Fig. 9:**
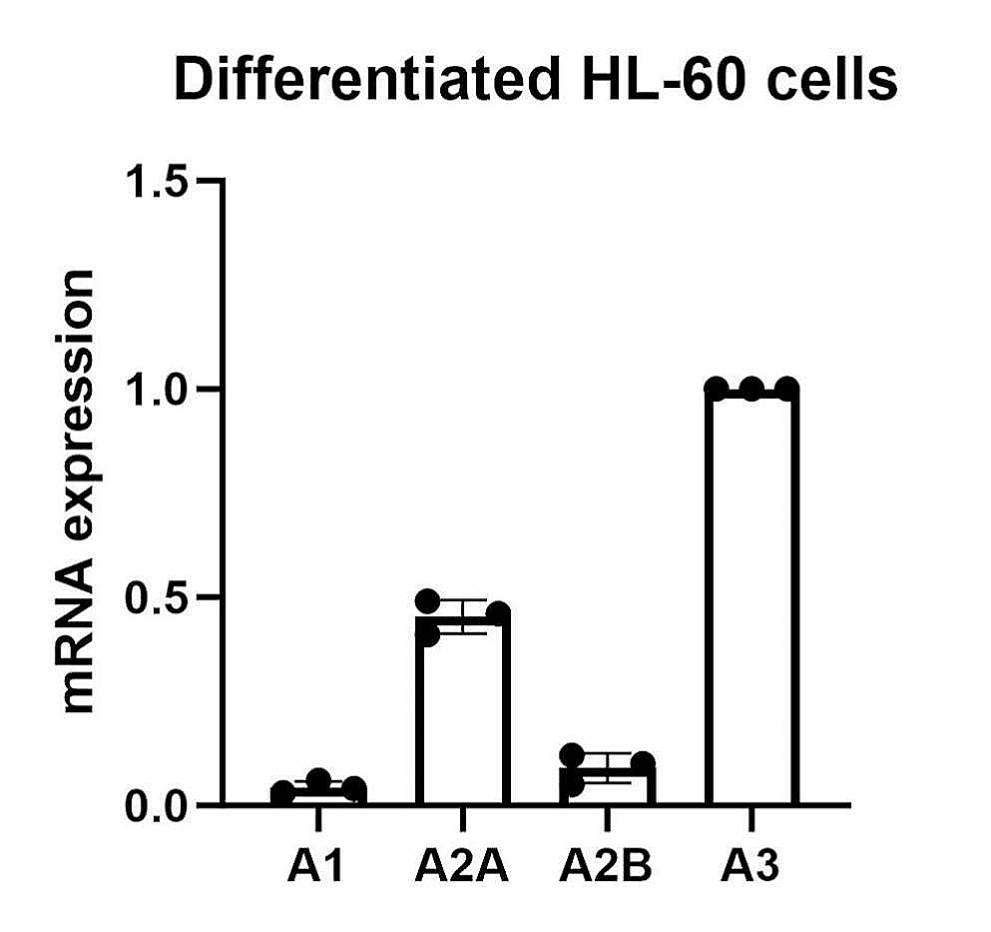
Gene expression levels of the four AR subtypes in differentiated HL-60 cells

Figure 9 shows that the A_3_AR is the highest expressed of the four AR subtypes in differentiated HL-60 cells. The A_2A_AR is also at a relatively high level but is only about 40% of the A_3_AR. The A_1_AR and A_2B_AR are less than 10% of the A_3_AR expression level. Thus, both the A_3_AR selectivity of MRS5698 and high A_3_AR expression in differentiated HL-60 cells guaranteed that the genetic and functional regulation by A_3_AR agonist occurs via the A_3_AR. The effects of the A_3_AR PAM showing changes in parallel to those of the A_3_AR agonist further supported this nature of regulation. Also, a study of off-target activities of a congeneric series of nucleoside/nucleobase derivatives, including MRS5698, indicated only weak µM affinity at a few other receptors [38].

## Discussion

The A_3_AR is known to play an essential role in various physiological processes, and its activation can influence gene regulation and various cellular responses [1,3,6,7). The present study showed that A_3_AR activation can lead to the upregulation of genes associated with both pro-inflammation and anti-inflammation, e.g. NFKB1Z and NFKB1A. The NF-κB response suppressor that also attenuates inflammation, aging, and cancer [32], may at least partially explain the anti-inflammatory effect of A_3_AR agonists. The upregulation of IL-1a and IL-1b could be related to the proinflammatory role of the A_3_AR.

The A_3_AR has been reported both pro- and anti-inflammatory depending on cell types [6, 7, 9, 10, 39]. For example, A_3_AR agonist Cl-IB-MECA (0.1 mg/kg, s.c.) has been found to induce an antiinflammatory and protective effect on the rat liver [39]. Wagner et al. [9] characterized the role of A_3_AR in a murine model of lung inflammation and found elevated pulmonary A_3_AR expression levels following lipopolysaccharide (LPS) exposure in vivo. LPS inhalation increased the accumulation of polymorphonuclear leukocytes (PMN) in wild-type and whole-body A_3_AR(^−/−^) mice in all lung compartments. Pretreatment with the selective A_3_AR agonist Cl-IB-MECA significantly decreased the migration of PMNs into the lung interstitium and alveolar air space of wild-type but not A_3_(^−/−^) mice. Ren et al. [10] showed that treatment of colonic mucosa from patients with ulcerative colitis (UC) with 100 nM Cl-IB-MECA for 24 h downregulated A_3_AR expression and upregulated TNF-α, NF-κB, and IL-1β production. A_3_AR activation by Cl-IB-MECA significantly decreased TNF-α and IL-1β production and attenuated the NF-κB p65 activation in colonic tissues from patients with colonic mucosal inflammation in UC. Hasko et al. [11] reported that in RAW 264.7 macrophages, A_3_AR agonist IB-MECA, at 0.2 and 0.5 mg/kg, potentiated LPS-stimulated IL-10 production and inhibited LPS-induced TNF-a production, thus suggesting anti-inflammatory action. Sajjadi et al. [12] reported that A_3_AR stimulation caused a marked decrease in TNF-α mRNA, but not IL-1b, IL-6, or IL-8 mRNA. Thus, the pro- and anti-inflammatory effects of adenosine and the A_3_AR are dependent on cell types.

The A_3_AR agonist modulates gene expression and is known to occur through various intracellular signaling pathways. The present study further analyzed pathways potentially involved in the A_3_AR function. Pathway analysis highlighted several important signaling pathways downstream of the A_3_AR, including IL-6, IL-17, and P38 MAPK signaling. UPS analysis of regulators from all three groups MRS5698 (G1-S-1-ipa), LUF6000 (G2-S-ipa), and MRS5698 + LUF6000 (G3-S-ipa) identified important regulatory molecules including IL-1, NFkB, JNK, and ERK. Analysis of known network interactions and regulated genes reveals interactions among many important signaling molecules upon A_3_AR activation, e.g. IL-1b, IL-17, TNF-α, and inflammation response.

IB-MECA and Cl-IB-MECA, which are moderately selective A_3_AR agonists, have been used in many earlier studies [11, 12, 40]. However, at a higher concentration, both agonists may activate other AR subtypes. For example. Kreckler et al. [41] reported that adenosine inhibits tumor necrosis factor-α release from mouse peritoneal macrophages via A_2A_ and A_2B_ but not the A_3_AR. Thus, the anti-inflammatory effect of adenosine is dependent on both AR subtypes in addition to cell types. Thus, to elucidate the role of the A_3_AR (not to activate other AR subtypes), we chose a more selective A_3_AR agonist MRS5698, and an A_3_AR allosteric enhancer LUF6000 in the present study.

A critical role of the A_3_AR in neutrophil behavior has been suggested [22, 42]. The A_3_AR is also highly expressed in monocyte/macrophage, dendritic cells, cytotoxic and helper T cells, and mast cells [6]. It has also been suggested that chronic treatment with an agonist-induced A_3_AR downregulation. This is possibly responsible for the suppression of its basal inhibitory effect on cytokine production. However, few if any studies have reported the impact of acute A_3_AR agonist treatment on genetic regulation.

Ochaion et al. [40] reported that A_3_AR agonist (1′*S*,2′*R*,3′*S*,4′*R*,5′*S*)-4-(2-chloro-6-(3-chlorobenzylamino)-9*H*-purin-9-yl)-2,3-dihydroxy-*N*-methylbicyclo[3.1.0]hexane-1-carboxamide (CF502, MRS3558), via de-regulation of the NF-kB signaling pathway, inhibits fibroblast-like synoviocyte (FLS) growth and the inflammatory manifestations of arthritis, supporting the development of A_3_AR agonists for the treatment of rheumatoid arthritis. The A_3_AR expression level was downregulated shortly after CF502 treatment. Subsequently, the expression levels of protein kinase B/Akt (PKB/Akt), NF-kB, and TNF-α decreased. However, by studying A_3_AR knockout mice, Inoue et al. [42] suggested that A_3_AR antagonists could improve the efficacy of hypertonic saline resuscitation by reducing side effects in patients whose polymorphonuclear neutrophils are activated before hypertonic saline treatment.

Németh et al. [16] hypothesized that adenosine may exert some of its anti-inflammatory effects by decreasing NF-kB activation because gene expression of most of the proinflammatory cytokines inhibited by adenosine is dependent on NF-kB activation. Microarray analyses revealed that mRNA levels of neither TNF-α nor other cytokines were altered by adenosine (100 µM) in either lipopolysaccharide (LPS)-stimulated, or non-stimulated RAW 264.7 macrophages. Although LPS induced expression of several other, noncytokine genes, such as A_2B_AR, adenosine did not affect the expression of these genes. Furthermore, adenosine (100 µM) as well as NECA and other agonists (10 µM) failed to decrease LPS-induced NF-κB DNA binding, NF-κB promoter activity, p65 nuclear translocation, and inhibitory κB degradation. Thus, the authors suggest that the anti-inflammatory effects of adenosine are independent of NF-κB [16]. Streitova et al. [43] showed mRNA expression for all four AR subtypes in normal and LPS-activated murine RAW 264.7 macrophages. LPS upregulated A_2A_ and A_2B_ mRNA expression, but downregulated A_3_AR. Ehrchen et al. [44] performed microarray analysis on glucocorticoid-treated monocytes and macrophages and identified glucocorticoid-dependent regulation of 133 genes, including anti-inflammatory A_3_AR, CD1d, and IL-1 receptor II. Barczyk et al. [45] reported that glucocorticoids promote the survival of anti-inflammatory macrophages via A_3_AR stimulation. Baram et al. [46] showed that by coupling to Gi3, the A_3_AR stimulates multiple signaling pathways in human mast cells, leading to upregulation of cytokines, chemokines, and growth factors, although it remains to be shown in neutrophils.

It is known that acute and chronic administration of A_3_AR agonists produced different functional or behavioral effects [47]. Considering that the A_3_AR-related gene downregulation based on chronic administration of Cl-IB-MECA has been recently reported [15], we observed that the total genes are almost equally up and downregulated in HL-60 cells treated with MRS5698 for 20 min. However, it is to be noted that the effects of Cl-IB-MECA were determined in mouse cells, and there are significant species-dependent differences in the pharmacology and role of the A_3_AR [14].

In summary, the present study identified genes and pathways that are regulated by the A_3_AR activation in a neutrophil model HL-60 cells, by using both a recently available and selective A_3_AR agonist, MRS5698, and the allosteric enhancer LUF6000. Based on the selectivity profile of both MRS5698 and LUF6000 together with the AR receptor expression profile of HL-60 cells, we are confident that the regulated genes and pathways are solely via the A_3_ARs. The MRS5698 + LUF6000 group had overall effects larger than MRS5698 alone, consistent with allosteric enhancement. We also observed the allosteric enhancement of the native A_3_AR function by LUF6000 in HL-60 cells. The patterns of transcriptional enhancement by LUF6000 are consistent with those of the functional enhancement. The findings from the present study support the role of A_3_AR in inflammation and cancer [6, 7, 48–50] and may shed light on the further development of A_3_AR agonists and allosteric enhancers for various conditions.

## Electronic supplementary material

Below is the link to the electronic supplementary material.

Supplementary Material 1

## Data Availability

The data that support the findings of this study are available from the corresponding authors upon reasonable request.

## Abbreviations

ANOVA: Analysis of Variance
AR: adenosine receptor
BWA: Burrows-Wheeler Aligner
GPCR: G protein-coupled receptor
HEPES: 4-(2-hydroxyethyl)-1-piperazineethanesulfonic acid
LPS: lipopolysaccharide
NF-κB: nuclear factor kappa-light-chain-enhancer of activated B cells
PAM: positive allosteric modulator
PMN: polymorphonuclear leukocytes
PPAR: peroxisome proliferator-activated receptor
RMA: Robust Multichip Average
UC: ulcerative colitis
WT: wild-type

## Declarations

### Inclusion and diversity

We support inclusive, diverse and equitable conduct of research.

## References

[1] IJzerman AP, Jacobson KA, Müller CE, Cronstein BN, Cunha RA (2022) International Union of Basic and Clinical Pharmacology. CXII: Adenosine receptors: a further update. Pharmacol Rev 74(2):340–372. 10.1124/pharmrev.121.000445 35302044 10.1124/pharmrev.121.000445 PMC8973513

[2] Chen JF, Eltzschig HK, Fredholm BB (2013) Adenosine receptors as drug targets–what are the challenges? Nat Rev Drug Discov 12(4):265–286. 10.1038/nrd3955 10.1038/nrd3955 PMC3930074 23535933

[3] Jacobson KA, Gao ZG (2006) Adenosine receptors as therapeutic targets. Nat Rev Drug Discov 5(3):247–264. 10.1038/nrd1983 16518376 10.1038/nrd1983 PMC3463109

[4] Linden J (2005) Adenosine in tissue protection and tissue regeneration. Mol Pharmacol 67(5):1385–1387. 10.1124/mol.105.011783 15703375 10.1124/mol.105.011783

[5] Jacobson KA, Tosh DK, Jain S, Gao ZG (2019) Historical and current adenosine receptor agonists in preclinical and clinical development. Front Cell Neurosci 13:124. 10.3389/fncel.2019.00124 30983976 10.3389/fncel.2019.00124 PMC6447611

[6] Jacobson KA, Merighi S, Varani K, Borea PA, Baraldi S, Aghazadeh Tabrizi M, Romagnoli R, Baraldi PG, Ciancetta A, Tosh DK, Gao ZG, Gessi S (2018) A_3_ adenosine receptors as modulators of inflammation: from medicinal chemistry to therapy. Med Res Rev 38(4):1031–1072. 10.1002/med.21456 28682469 10.1002/med.21456 PMC5756520

[7] Borea PA, Varani K, Vincenzi F, Baraldi PG, Tabrizi MA, Merighi S, Gessi (2015) The A_3_ adenosine receptor: history and perspectives. Pharmacol Rev 67(1):74–102 25387804 10.1124/pr.113.008540

[8] Gessi S, Merighi S, Varani K, Leung E, Mac Lennan S, Borea PA (2008) The A_3_ adenosine receptor: an enigmatic player in cell biology. Pharmacol Ther 117(1):123–140. 10.1016/j.pharmthera.2007.09.002 18029023 10.1016/j.pharmthera.2007.09.002

[9] Wagner R, Ngamsri KC, Stark S, Vollmer I, Reutershan J (2010) Adenosine receptor A_3_ is a critical mediator in LPS-induced pulmonary inflammation. Am J Physiol Lung Cell Mol Physiol 299(4):L502–512. 10.1152/ajplung.00083.2010 20639349 10.1152/ajplung.00083.2010

[10] Ren TH, Lv MM, An XM, Leung WK, Seto WK (2020) Activation of adenosine A_3_ receptor inhibits inflammatory cytokine production in colonic mucosa of patients with ulcerative colitis by downregulating the nuclear factor-kappa B signaling. J Dig Dis 21(1):38–45. 10.1111/1751-2980.12831 31714673 10.1111/1751-2980.12831

[11] Haskó G, Szabó C, Németh ZH, Kvetan V, Pastores SM, Vizi ES (1996) Adenosine receptor agonists differentially regulate IL-10, TNF-alpha, and nitric oxide production in RAW 264.7 macrophages and in endotoxemic mice. J Immunol 157(10):4634–4640 8906843 10.4049/jimmunol.157.10.4634

[12] Sajjadi FG, Takabayashi K, Foster AC, Domingo RC, Firestein GS (1996) Inhibition of TNF-alpha expression by adenosine: role of A_3_ adenosine receptors. J Immunol 156(9):3435–34342 8617970 10.4049/jimmunol.156.9.3435

[13] Cohen S, Barer F, Bar-Yehuda S, IJzerman AP, Jacobson KA, Fishman P (2014) A_3_ adenosine receptor allosteric modulator induces an anti-inflammatory effect: in vivo studies and molecular mechanism of action. Mediators Inflamm 2014:708746. 10.1155/2014/708746 25374446 10.1155/2014/708746 PMC4211160

[14] Gao ZG, Auchampach JA, Jacobson KA (2023) Species dependence of A_3_ adenosine receptor pharmacology and function. Purinergic Signal 19(3):523–550. 10.1007/s11302-022-09910-1 36538251 10.1007/s11302-022-09910-1 PMC9763816

[15] Lillo A, Serrano-Marín J, Lillo J, Raïch I, Navarro G, Franco R (2023) Gene regulation in activated microglia by adenosine A_3_ receptor agonists: a transcriptomics study. Purinergic Signal. Jan 27. 10.1007/s11302-022-09916-9 10.1007/s11302-022-09916-9 PMC11189369 36703008

[16] Németh ZH, Leibovich SJ, Deitch EA, Vizi ES, Szabó C, Hasko G (2003) cDNA microarray analysis reveals a nuclear factor-kappab-independent regulation of macrophage function by adenosine. J Pharmacol Exp Ther 306(3):1042–1049. 10.1124/jpet.103.052944 12766259 10.1124/jpet.103.052944

[17] Lillo A, Serrano-Marín J, Lillo J, Raïch I, Navarro G, Franco R (2023) Differential gene expression in activated microglia treated with adenosine A_2A_ receptor antagonists highlights olfactory receptor 56 and T-cell activation GTPase-activating protein 1 as potential biomarkers of the polarization of activated microglia. Cells 12:2213. 10.3390/cells12182213 37759436 10.3390/cells12182213 PMC10526142

[18] Kolaczkowska E, Kubes P (2013) Neutrophil recruitment and function in health and inflammation. Nat Rev Immunol 13:159–175. 10.1038/nri3399 23435331 10.1038/nri3399

[19] Van Haastert PJ, Devreotes PN (2004) Chemotaxis: signalling the way forward. Nat Rev Mol Cell Biol 5:626–634. 10.1038/nrm1435 15366706 10.1038/nrm1435

[20] Hauert AB, Martinelli S, Marone C, Niggli V, Differentiated (2002) HL-60 cells are a valid model system for the analysis of human neutrophil migration and chemotaxis. Int J Biochem Cell Biol 34:838–854. 10.1016/s1357-2725(02)00010-9 11950599 10.1016/s1357-2725(02)00010-9

[21] Woo CH, Yoo MH, You HJ, Cho SH, Mun YC, Seong CM, Kim JH (2003) Transepithelial migration of neutrophils in response to leukotriene B4 is mediated by a reactive oxygen species-extracellular signal-regulated kinase-linked cascade. J Immunol 170:6273–6279. 10.4049/jimmunol.170.12.6273 12794160 10.4049/jimmunol.170.12.6273

[22] Chen Y, Corriden R, Inoue Y, Yip L, Hashiguchi N, Zinkernagel A, Nizet V, Insel PA, Junger WG (2006) ATP release guides neutrophil chemotaxis via P2Y_2_ and A_3_ receptors. Science 314(5806):1792–1795. 10.1126/science.1132559 17170310 10.1126/science.1132559

[23] Carrigan SO, Pink DB, Stadnyk AW (2007) Neutrophil transepithelial migration in response to the chemoattractant fMLP but not C5a is phospholipase D-dependent and related to the use of CD11b/CD18. J Leukoc Biol 82:1575–1584. 10.1189/jlb.0806528 17724165 10.1189/jlb.0806528

[24] Kohno Y, Sei Y, Koshiba M, Kim HO, Jacobson KA (1996) Induction of apoptosis in HL-60 human promyelocytic leukemia cells by selective adenosine A_3_ receptor agonists. Biochem Biophys Res Comm. ;219:904–910. Correction: 1996;221:849 10.1006/bbrc.1996.0331 PMC4553948 8645277

[25] Gessi S, Varani K, Merighi S, Cattabriga E, Iannotta V, Leung E, Baraldi PG, Borea PA (2002) A_3_ adenosine receptors in human neutrophils and promyelocytic HL60 cells: a pharmacological and biochemical study. Mol Pharmacol 61(2):415–424. 10.1124/mol.61.2.415 11809867 10.1124/mol.61.2.415

[26] Koscsó B, Csóka B, Pacher P, Haskó G (2011) Investigational A_3_ adenosine receptor targeting agents. Expert Opin Investig Drugs 20(6):757–768. 10.1517/13543784.2011.573785 10.1517/13543784.2011.573785 PMC3613226 21457061

[27] Tosh DK, Padia J, Salvemini D, Jacobson KA (2015) Efficient, large-scale synthesis and preclinical studies of MRS5698, a highly selective A_3_ adenosine receptor agonist that protects against chronic neuropathic pain. Purinergic Signal 11:371–387 26111639 10.1007/s11302-015-9459-2 PMC4529848

[28] Göblyös A, Gao ZG, Brussee J, Connestari R, Neves Santiago S, Ye K, IJzerman AP, Jacobson KA (2006) Structure activity relationships of 1*H*-imidazo[4,5-*c*]quinolin-4-amine derivatives new as allosteric enhancers of the A_3_ adenosine receptor. J Med Chem 49:3354–3361 16722654 10.1021/jm060086s PMC2547348

[29] Gao ZG, Levitan IM, Inoue A, Wei Q, Jacobson KA (2023) A_2B_ adenosine receptor activation and modulation by protein kinase C. iScience 26(7):107178. 10.1016/j.isci.2023.107178 37404375 10.1016/j.isci.2023.107178 PMC10316653

[30] Gao ZG, Verzijl D, Zweemer A, Ye K, Göblyös A, Ijzerman AP, Jacobson KA (2011) Functionally biased modulation of A_3_ adenosine receptor agonist efficacy and potency by imidazoquinolinamine allosteric enhancers. Biochem Pharmacol 82(6):658–668. 10.1016/j.bcp.2011.06.017 21718691 10.1016/j.bcp.2011.06.017 PMC3152598

[31] Totzke G, Essmann F, Pohlmann S, Lindenblatt C, Jänicke RU, Schulze-Osthoff K (2006) A novel member of the IkappaB family, human IkappaB-zeta, inhibits transactivation of p65 and its DNA binding. J Biol Chem 281(18):12645–12654. 10.1074/jbc.M511956200 16513645 10.1074/jbc.M511956200

[32] Cartwright T, Perkins ND, Wilson L (2016) NFKB1: a suppressor of inflammation, ageing and cancer. FEBS J 283(10):1812–1822. 10.1111/febs.13627 26663363 10.1111/febs.13627

[33] Giordano M, Roncagalli R, Bourdely P, Chasson L, Buferne M, Yamasaki S, Beyaert R, van Loo G, Auphan-Anezin N, Schmitt-Verhulst AM, Verdeil G (2014) The tumor necrosis factor alpha-induced protein 3 (TNFAIP3, A20) imposes a brake on antitumor activity of CD8 T cells. Proc Natl Acad Sci USA 111(30):11115–11120. 10.1073/pnas.1406259111 25024217 10.1073/pnas.1406259111 PMC4121810

[34] Lee D, Shin KJ, Kim DW, Yoon KA, Choi YJ, Lee BNR, Cho JY (2018) CCL4 enhances preosteoclast migration and its receptor CCR5 downregulation by RANKL promotes osteoclastogenesis. Cell Death Dis 9(5):495. 10.1038/s41419-018-0562-5 29717113 10.1038/s41419-018-0562-5 PMC5931580

[35] Tsai CH, Lai AC, Lin YC, Chi PY, Chen YC, Yang YH, Chen CH, Shen SY, Hwang TL, Su MW, Hsu IL, Huang YC, Maitland-van der Zee AH, McGeachie MJ, Tantisira KG, Chang YJ, Lee YL (2023) Neutrophil extracellular trap production and CCL4L2 expression influence corticosteroid response in asthma. Sci Transl Med 15(699):eadf3843. 10.1126/scitranslmed.adf3843 37285400 10.1126/scitranslmed.adf3843

[36] Kim YH, Lee EE, Sim HW, Kang EK, Won YH, Lee DE, Hong KM, Song YW (2021) CCL3L3-null status is associated with susceptibility to systemic lupus erythematosus. Sci Rep 11(1):19172. 10.1038/s41598-021-98531-6 34580371 10.1038/s41598-021-98531-6 PMC8476559

[37] Du L, Gao ZG, Nithipatikom K, IJzerman AP, Veldhoven JP, Jacobson KA, Gross GJ, Auchampach JA (2012) Protection from myocardial ischemia/reperfusion injury by a positive allosteric modulator of the A_3_ adenosine receptor. J Pharmacol Exp Ther 340(1):210–217. 10.1124/jpet.111.187559 22011434 10.1124/jpet.111.187559 PMC3251031

[38] Paoletta S, Tosh DK, Salvemini D, Jacobson KA (2014) Structural probing of off-target G protein-coupled receptor activities within a series of adenosine/adenine congeners. PLoS ONE 9:e97858. 10.1371/journal.pone.0097858 24859150 10.1371/journal.pone.0097858 PMC4032265

[39] Ohana G, Cohen S, Rath-Wolfson L, Fishman P (2016) A_3_ adenosine receptor agonist, CF102, protects against hepatic ischemia/reperfusion injury following partial hepatectomy. Mol Med Rep 14(5):4335–4341. 10.3892/mmr.2016.5746 27666664 10.3892/mmr.2016.5746

[40] Ochaion A, Bar-Yehuda S, Cohen S, Amital H, Jacobson KA, Joshi BV, Gao ZG, Barer F, Patoka R, Del Valle L, Perez-Liz G, Fishman P (2008) The A_3_ adenosine receptor agonist CF502 inhibits the PI3K, PKB/Akt and NF-kappaB signaling pathway in synoviocytes from rheumatoid arthritis patients and in adjuvant-induced arthritis rats. Biochem Pharmacol 76(4):482–494. 10.1016/j.bcp.2008.05.032 18602896 10.1016/j.bcp.2008.05.032 PMC2677448

[41] Kreckler LM, Wan TC, Ge ZD, Auchampach JA (2006) Adenosine inhibits tumor necrosis factor-alpha release from mouse peritoneal macrophages via A_2A_ and A_2B_ but not the A_3_ adenosine receptor. J Pharmacol Exp Ther 317(1):172–180. 10.1124/jpet.105.096016 16339914 10.1124/jpet.105.096016

[42] Inoue Y, Chen Y, Hirsh MI, Yip L, Junger WG (2008) A_3_ and P2Y_2_ receptors control the recruitment of neutrophils to the lungs in a mouse model of sepsis. Shock 30:173–177 18091570 10.1097/SHK.0b013e318160dad4 PMC4212521

[43] Štreitová D, Hofer M, Holá J, Vacek A, Pospíšil M (2010) Adenosine A_1_, A_2a_, A_2b_, and A_3_ receptors in hematopoiesis. 2. Expression of receptor mRNA in resting and lipopolysaccharide-activated mouse RAW 264.7 macrophages. Physiol Res 59(1):139–144. 10.33549/physiolres.931724 19249906 10.33549/physiolres.931724

[44] Ehrchen J, Steinmüller L, Barczyk K, Tenbrock K, Nacken W, Eisenacher M, Nordhues U, Sorg C, Sunderkötter C, Roth J (2007) Glucocorticoids induce differentiation of a specifically activated, anti-inflammatory subtype of human monocytes. Blood 109(3):1265–1274. 10.1182/blood-2006-02-001115 17018861 10.1182/blood-2006-02-001115

[45] Barczyk K, Ehrchen J, Tenbrock K, Ahlmann M, Kneidl J, Viemann D, Roth J (2010) Glucocorticoids promote survival of anti-inflammatory macrophages via stimulation of adenosine receptor A_3_. Blood 116(3):446–455. 10.1182/blood-2009-10-247106 20460503 10.1182/blood-2009-10-247106

[46] Baram D, Dekel O, Mekori YA, Sagi-Eisenberg R (2010) Activation of mast cells by trimeric G protein Gi3; coupling to the A_3_ adenosine receptor directly and upon T cell contact. J Immunol 184(7):3677–3688. 10.4049/jimmunol.0901333Epub 2010 Feb 26 20190146 10.4049/jimmunol.0901333

[47] Jacobson KA (1998) Adenosine A_3_ receptors: novel ligands and paradoxical effects. Trends Pharmacol Sci 19(5):184–191. 10.1016/s0165-6147(98)01203-6 9652191 10.1016/s0165-6147(98)01203-6 PMC3158240

[48] Ledderose C, Hefti MM, Chen Y, Bao Y, Seier T, Li L, Woehrle T, Zhang J, Junger WG (2016) Adenosine arrests breast cancer cell motility by A_3_ receptor stimulation. Purinergic Signal 12:673–685 27577957 10.1007/s11302-016-9531-6 PMC5124008

[49] Notarbartolo M, Lo Cicero S, Meli M, Poma P, Labbozzetta M, Cervello M, D’Alessandro N (2005) Induction of apoptosis by the adenosine derivative IB-MECA in parental or multidrug-resistant HL-60 leukemia cells: possible relationship to the effects on inhibitor of apoptosis protein levels. Chemotherapy 51(5):272–279. 10.1159/000087255 16088125 10.1159/000087255

[50] Li S, Huang S, Peng SB (2005) Overexpression of G protein-coupled receptors in cancer cells: involvement in tumor progression. Int J Oncol 27(5):1329–1339 16211229

