# Supplementary material for: Genetic and functional modulation by agonist MRS5698 and allosteric enhancer LUF6000 at the native A3 adenosine receptor in HL-60 cells
Source: Purinergic Signal. 2024 Feb 28;20(5):559–70. doi: 10.1007/s11302-024-09992-z (PMC11377395; doi:10.1007/s11302-024-09992-z)
Supplement: Supplementary file 1 — Supplementary Material 1 [file 11302_2024_9992_MOESM1_ESM.docx]

**Supporting information file**

Genetic and functional modulation by agonist MRS5698 and allosteric enhancer LUF6000

at the native A_3_ adenosine receptor in HL-60 cells

Zhan-Guo Gao,^a,^* Weiping Chen,^b^ Ray R. Gao,^b^ Jonathan Li,^b^ Dilip K. Tosh,^a^ John A. Hanover,^b^ Kenneth A. Jacobson^a,^*

^a^Molecular Recognition Section, Laboratory of Bioorganic Chemistry, NIDDK, National Institutes of Health, 9000 Rockville Pike, Bethesda, MD 20892, USA.

^b^Genomics Core, NIDDK, National Institutes of Health, 9000 Rockville Pike, Bethesda, MD 20892, USA.

Contents page

**Figure S1**. Affected pathways in differentiated HL-60 cells S2 – S3

**Figure S2**. List of upstream regulators from IPA analysis S4 – S6

**Table S1**. Genes regulated by A_3_AR activation

in differentiated HL-60 cells S7 – S20

**Figure S1**. A complete list of affected pathways in differentiated HL-60 cells, from the IPA analysis. The treatment groups are: MRS5698 (G1-S-1-ipa), LUF6000 (G2-S-ipa), MRS5698+LUF6000 (G3-S-ipa).


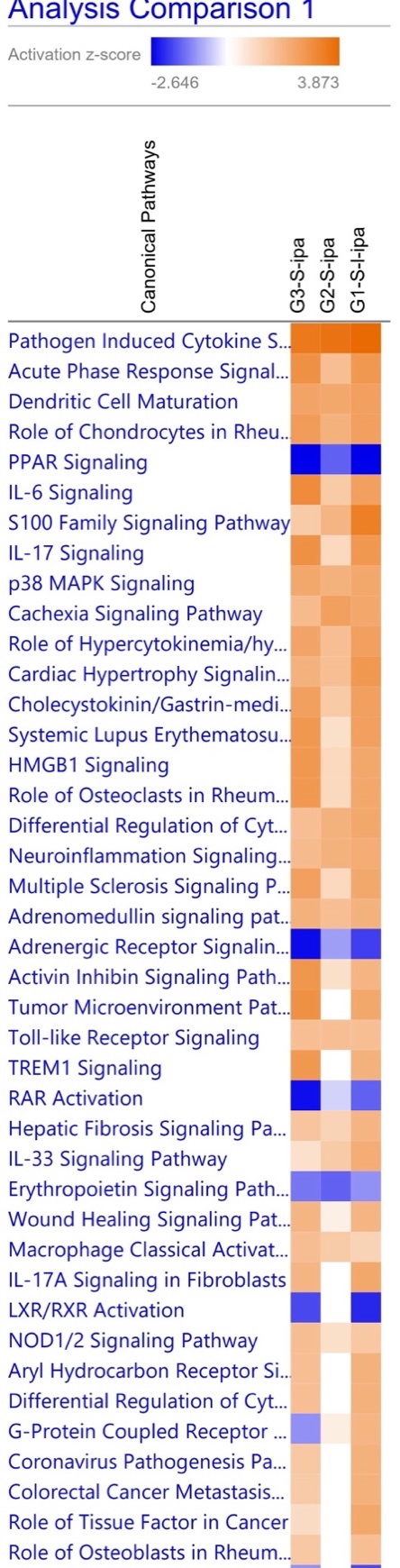


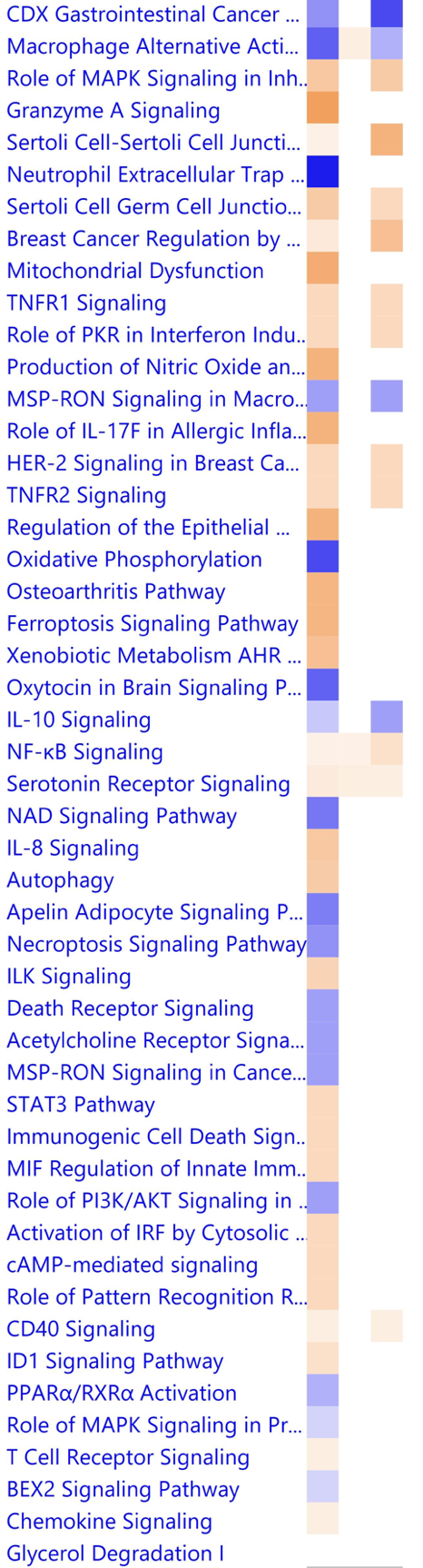


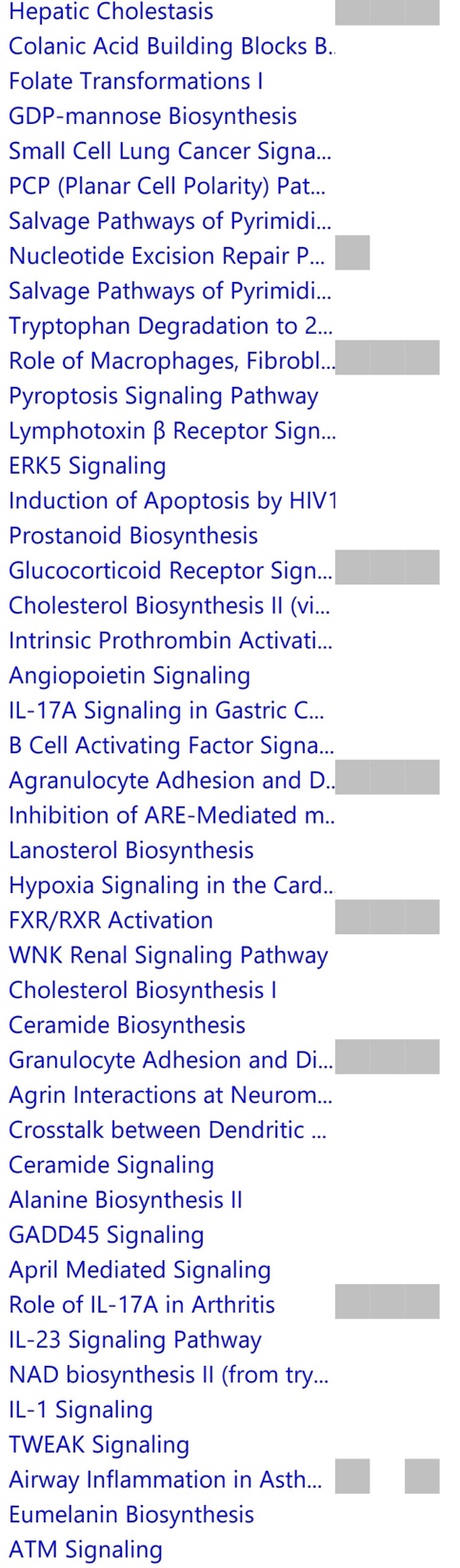


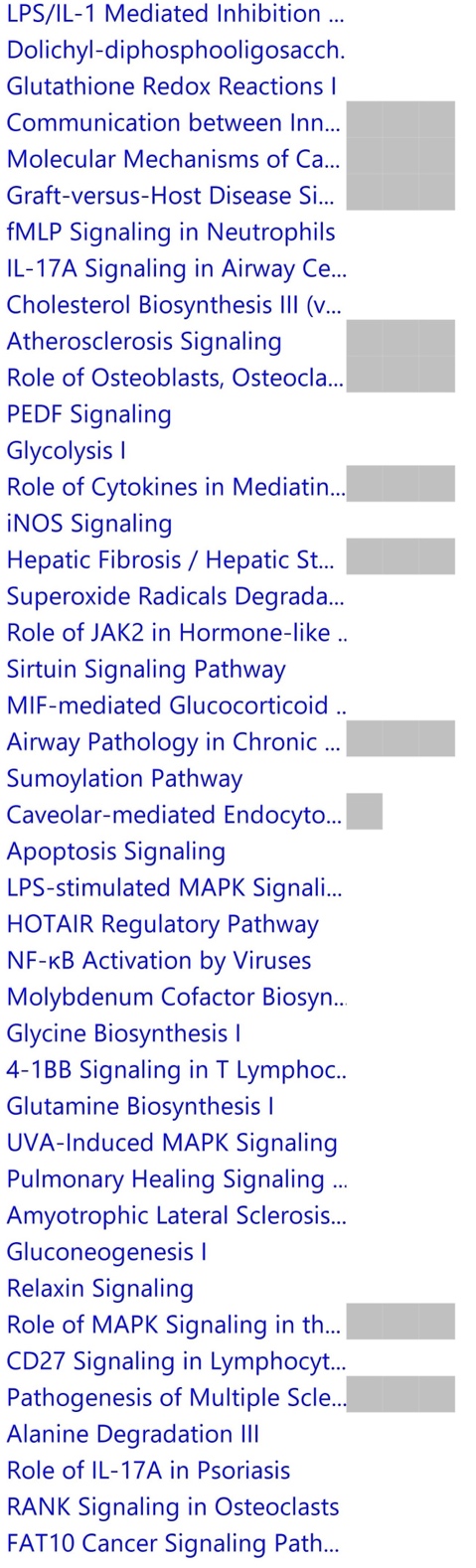


**Figure S2**. The more complete list of upstream regulators from IPA analysis (genes showing insignificant regulation are not shown). The treatment groups are: MRS5698 (G1-S-1-ipa), LUF6000 (G2-S-ipa), MRS5698+LUF6000 (G3-S-ipa).


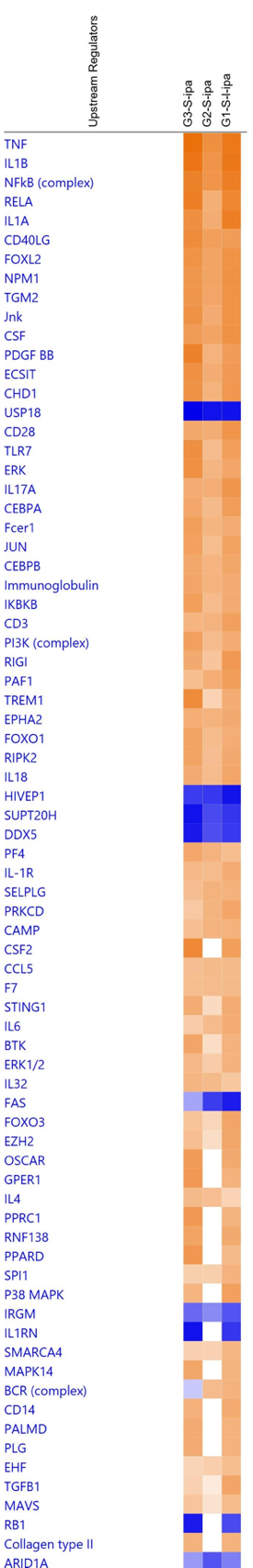


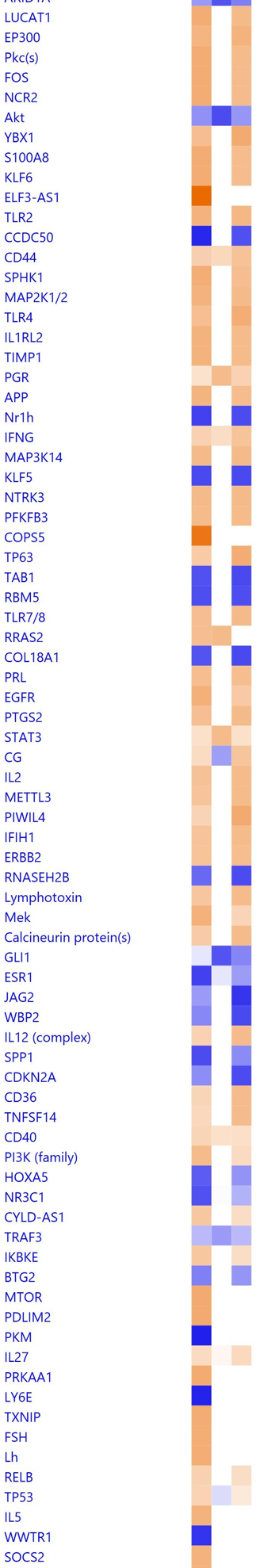


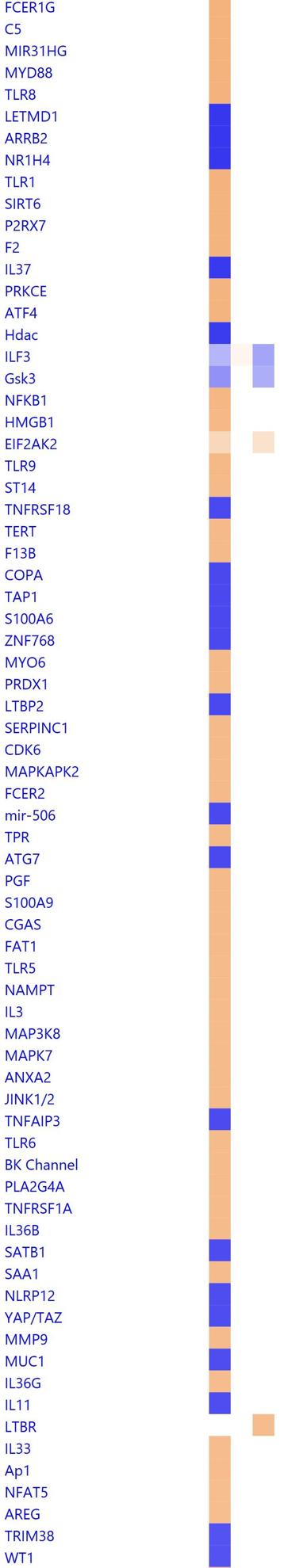


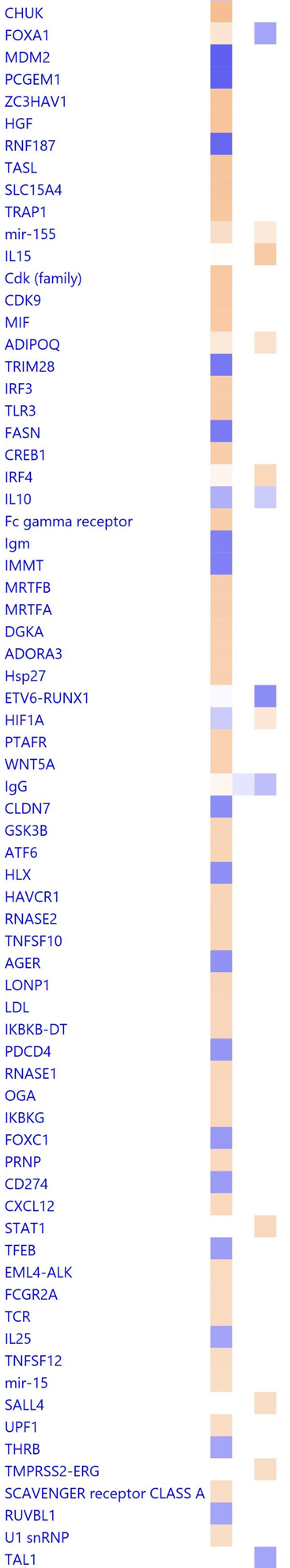


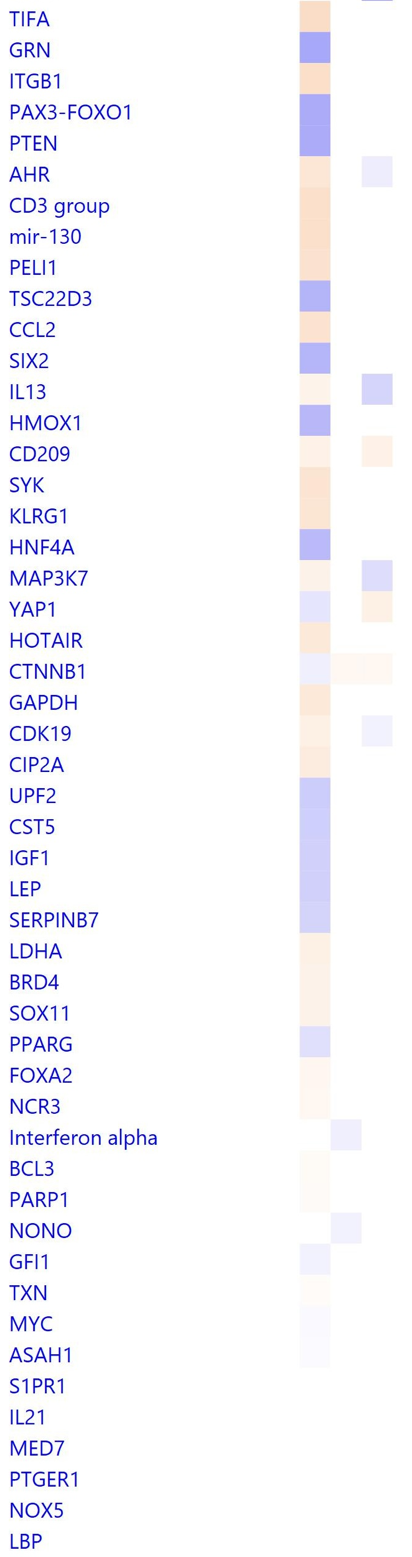


**Table S1**. The complete list of genes regulated by A_3_AR activation in differentiated HL-60 cells. Treatment groups are MRS5698 (G1), LUF6000 (G2), and MRS5698+LUF6000 (G3). CK is control (untreated) cells; FDR represents a statistical threshold.

| Gene name | P-value (G1 vs CK) | FDR step up (G1 vs CK) | Fold change (G1 vs CK) | P-value (G2 vs CK) | FDR step up (G2 vs CK) | Fold change (G2 vs CK) | P-value (1+2 vs CK) | FDR step up (1+2 vs CK) | Fold change (1+2 vs CK) | Number of occurrences |
| --- | --- | --- | --- | --- | --- | --- | --- | --- | --- | --- |
| TNFAIP3 | 5.73E-10 | 8.65E-06 | 5.06991 | 4.09E-07 | 0.00617298 | 2.02543 | 1.01E-10 | 1.52E-06 | 7.53548 | 3 |
| IL1B | 6.33E-08 | 0.0003037 | 42.962 | 7.61E-06 | 0.0383136 | 7.58597 | 4.15E-08 | 0.00018109 | 52.499 | 3 |
| NFKBIA | 8.51E-08 | 0.0003037 | 3.36089 | 5.60E-06 | 0.0383136 | 2.03344 | 4.17E-08 | 0.00018109 | 3.77919 | 3 |
| CCL4 | 9.49E-08 | 0.0003037 | 5.85742 | 2.72E-05 | 0.102633 | 2.29939 | 7.76E-08 | 0.00019534 | 6.14125 | 3 |
| CCL4L2 | 1.01E-07 | 0.0003037 | 7.72217 | 4.02E-05 | 0.11132 | 2.51109 | 4.81E-08 | 0.00018109 | 9.46755 | 3 |
| IL1A | 1.61E-07 | 0.00040455 | 56.7231 | 0.00011727 | 0.191918 | 5.67512 | 6.00E-08 | 0.00018109 | 98.6383 | 3 |
| CCL3L3 | 3.39E-07 | 0.00073189 | 6.19792 | 0.0001477 | 0.202795 | 2.23355 | 2.40E-07 | 0.00045281 | 6.72381 | 3 |
| IL1RN | 5.36E-07 | 0.0010119 | 7.80193 | 0.00204859 | 0.992848 | 1.95052 | 1.44E-07 | 0.00031074 | 11.4437 | 3 |
| IER3-AS1 | 9.85E-07 | 0.00165268 | 8.63167 | 0.00032503 | 0.327259 | 2.6344 | 4.45E-07 | 0.00064839 | 11.0316 | 3 |
| NFKBIZ | 1.37E-06 | 0.00207109 | 15.7143 | 7.86E-05 | 0.169661 | 4.87716 | 6.89E-07 | 0.00086699 | 20.4113 | 3 |
| EGR2 | 1.58E-06 | 0.00217401 | 4.05548 | 0.299249 | 0.997755 | 1.12005 | 4.72E-07 | 0.00064839 | 5.18766 | 2 |
| CCL3 | 3.94E-06 | 0.00495955 | 5.33071 | 0.00074676 | 0.66343 | 2.20141 | 5.82E-06 | 0.00439574 | 4.91808 | 3 |
| BIRC3 | 5.27E-06 | 0.00550103 | 3.15567 | 0.00010177 | 0.191918 | 2.17838 | 8.20E-07 | 0.00095231 | 4.33679 | 3 |
| ZFP36 | 5.32E-06 | 0.00550103 | 5.62784 | 0.00311249 | 0.997755 | 2.02633 | 4.48E-06 | 0.00399526 | 5.81506 | 3 |
| CXCL2 | 5.63E-06 | 0.00550103 | 10.795 | 0.00835603 | 0.997755 | 2.24551 | 2.13E-06 | 0.0022977 | 15.0916 | 3 |
| IER3 | 5.83E-06 | 0.00550103 | 7.41336 | 0.00106489 | 0.738299 | 2.54757 | 2.53E-06 | 0.00254664 | 9.44298 | 3 |
| PRDM1 | 7.43E-06 | 0.00660174 | 3.72453 | 0.00142186 | 0.894766 | 1.87939 | 4.50E-06 | 0.00399526 | 4.12028 | 3 |
| CXCL8 | 9.03E-06 | 0.00757876 | 1.78051 | 0.0103452 | 0.997755 | 1.21386 | 5.19E-06 | 0.00412223 | 1.866 | 1 |
| CCL3-AS1 | 1.62E-05 | 0.0128694 | 4.93978 | 0.00238141 | 0.992848 | 2.12127 | 3.15E-05 | 0.0164278 | 4.34024 | 3 |
| PTGS2 | 2.53E-05 | 0.0177945 | 2.5653 | 0.0269637 | 0.997755 | 1.34977 | 3.99E-07 | 0.00064839 | 5.10326 | 2 |
| SGK1 | 2.56E-05 | 0.0177945 | 4.3298 | 0.00600546 | 0.997755 | 1.8549 | 4.01E-05 | 0.0183634 | 4.02008 | 3 |
| G0S2 | 3.26E-05 | 0.0209079 | 4.4471 | 0.23557 | 0.997755 | 1.25442 | 8.23E-06 | 0.00565002 | 6.0404 | 2 |
| SOD2 | 3.32E-05 | 0.0209079 | 3.1457 | 0.014031 | 0.997755 | 1.53747 | 2.29E-05 | 0.0144408 | 3.43077 | 2 |
| JUN | 6.95E-05 | 0.0420102 | 2.05929 | 0.00981885 | 0.997755 | 1.38388 | 5.07E-06 | 0.00412223 | 2.80446 | 2 |
| CCRL2 | 0.00016287 | 0.0911068 | 5.71019 | 0.0128409 | 0.997755 | 2.38722 | 8.62E-05 | 0.031739 | 6.81738 | 3 |
| TSPAN11 | 0.0001694 | 0.0913718 | 2.09262 | 0.325479 | 0.997755 | 1.13626 | 0.00023664 | 0.0538405 | 2.02154 | 2 |
| PHLDA1-AS1 | 0.00027297 | 0.137422 | 2.55668 | 0.0177793 | 0.997755 | 1.63181 | 0.00011479 | 0.0346743 | 2.89246 | 2 |
| TNF | 0.00034565 | 0.168398 | 2.95647 | 0.391736 | 0.997755 | 1.14263 | 0.00046114 | 0.0696894 | 2.79671 | 2 |
| BTG2 | 0.00061104 | 0.279654 | 2.15987 | 0.68671 | 0.997755 | 1.05967 | 6.91E-05 | 0.0289835 | 2.85843 | 2 |
| PHLDA1 | 0.00065619 | 0.291483 | 2.2094 | 0.0454685 | 0.997755 | 1.45539 | 0.00029605 | 0.0612489 | 2.43832 | 2 |
| CD83 | 0.00068453 | 0.295386 | 1.79299 | 0.0864345 | 0.997755 | 1.25985 | 4.01E-05 | 0.0183634 | 2.42352 | 1 |
| CCL20 | 0.00102121 | 0.385582 | 2.85805 | 0.0499968 | 0.997755 | 1.63886 | 8.27E-05 | 0.0312371 | 4.49279 | 2 |
| CLEC4E | 0.00104959 | 0.386632 | 4.39658 | 0.0772108 | 0.997755 | 2.04763 | 0.00070871 | 0.0861162 | 4.85945 | 2 |
| DDIT4 | 0.00200219 | 0.521364 | 1.7955 | 0.0212763 | 0.997755 | 1.44835 | 0.00023059 | 0.0535789 | 2.22367 | 1 |
| FAM78A | 0.00285389 | 0.653065 | -1.62948 | 0.00527625 | 0.997755 | -1.52107 | 7.35E-05 | 0.0297022 | -2.34067 | 1 |
| SPP1 | 0.00290211 | 0.654187 | 1.59108 | 0.0682408 | 0.997755 | 1.26527 | 0.00015334 | 0.0402951 | 2.10735 | 1 |
| AFMID | 0.00352487 | 0.737413 | -1.87358 | 0.0103334 | 0.997755 | -1.67537 | 0.00223573 | 0.114633 | -1.93736 | 2 |
| PDE4B | 0.00360015 | 0.737413 | 2.20475 | 0.475857 | 0.997755 | 1.14327 | 0.00153329 | 0.107209 | 2.56194 | 2 |
| ARHGAP33 | 0.00390744 | 0.73853 | 2.67166 | 0.425246 | 0.997755 | 1.19964 | 0.00287229 | 0.119505 | 2.91741 | 2 |
| GLMP | 0.00431984 | 0.776698 | -1.96366 | 0.338291 | 0.997755 | -1.19011 | 0.00637542 | 0.143764 | -1.88926 | 2 |
| IL6 | 0.00496251 | 0.823614 | 1.72025 | 0.0185917 | 0.997755 | 1.53466 | 0.00035528 | 0.0646482 | 2.34777 | 1 |
| PRR7 | 0.00554374 | 0.854357 | 2.60286 | 0.227428 | 0.997755 | 1.47985 | 0.00512545 | 0.136683 | 2.64192 | 2 |
| NOTCH4 | 0.00566458 | 0.863728 | 2.00459 | 0.895649 | 0.997755 | 1.02857 | 0.00038238 | 0.0684099 | 2.99624 | 2 |
| PIM3 | 0.00579512 | 0.863728 | 1.48493 | 0.0937877 | 0.997755 | 1.21511 | 0.00041828 | 0.0686966 | 1.82267 | 1 |
| ATN1 | 0.00583329 | 0.863728 | -1.32544 | 0.00641718 | 0.997755 | -1.32097 | 4.87E-05 | 0.0210225 | -1.8277 | 1 |
| IL23A | 0.00667114 | 0.941628 | 1.96509 | 0.044912 | 0.997755 | 1.57573 | 0.00423196 | 0.130631 | 2.04824 | 2 |
| PRKCSH | 0.00759459 | 0.980351 | -1.44309 | 0.0423217 | 0.997755 | -1.28948 | 0.00010486 | 0.0323208 | -2.06937 | 1 |
| GRIK1 | 0.009342 | 0.986062 | 1.60416 | 0.0249914 | 0.997755 | 1.46258 | 0.00080801 | 0.0861162 | 2.02243 | 1 |
| TRIM14 | 0.0109178 | 0.986062 | -1.71248 | 0.0115561 | 0.997755 | -1.71473 | 0.00354484 | 0.130631 | -1.99636 | 1 |
| FLII | 0.0117871 | 0.986062 | -1.42368 | 0.0396737 | 0.997755 | -1.31117 | 8.94E-05 | 0.0321543 | -2.15426 | 1 |
| IL17C | 0.012094 | 0.986062 | 1.83412 | 0.18546 | 0.997755 | 1.29022 | 0.00425533 | 0.130631 | 2.11921 | 2 |
| RSPH6A | 0.0126211 | 0.986062 | 1.50208 | 0.433225 | 0.997755 | 1.09645 | 0.00098733 | 0.0931947 | 1.92611 | 1 |
| --- | 0.998898 | 0.999795 | -1.06324 | 0.86577 | 0.997755 | -1.02097 | 0.927576 | 0.973909 | -1.08904 | 56 |
| CD4 | 0.014575 | 0.986062 | -1.45161 | 0.156099 | 0.997755 | -1.21337 | 0.00018426 | 0.0471679 | -2.14995 | 1 |
| LACC1 | 0.0147364 | 0.986062 | 1.50571 | 0.0226335 | 0.997755 | 1.49007 | 0.00207934 | 0.114633 | 1.80459 | 1 |
| MAFB | 0.0149645 | 0.986062 | 2.60073 | 0.116256 | 0.997755 | 1.85524 | 0.00686421 | 0.143764 | 3.1386 | 2 |
| MFSD4B | 0.0159959 | 0.986062 | 1.40256 | 0.00183924 | 0.992848 | 1.6644 | 0.00040567 | 0.0686966 | 1.89352 | 1 |
| SNORA14B | 0.0160204 | 0.986062 | 1.71117 | 0.728659 | 0.997755 | 1.07251 | 0.00425875 | 0.130631 | 2.09397 | 1 |
| TTC32 | 0.0172665 | 0.986062 | 2.17679 | 0.0353653 | 0.997755 | 1.89859 | 0.0277362 | 0.225351 | 1.95283 | 3 |
| RASGEF1B | 0.0176981 | 0.986062 | 1.88038 | 0.261172 | 0.997755 | 1.22397 | 0.0108739 | 0.16092 | 2.05712 | 2 |
| FAM223B | 0.0177564 | 0.986062 | 1.79209 | 0.0541808 | 0.997755 | 1.57605 | 0.00350311 | 0.129994 | 2.24053 | 1 |
| CDCA5 | 0.0193618 | 0.986062 | -1.67416 | 0.0630261 | 0.997755 | -1.48094 | 0.00768593 | 0.149012 | -1.91122 | 1 |
| CNN2 | 0.0222747 | 0.986062 | -1.72881 | 0.118187 | 0.997755 | -1.39006 | 0.00143403 | 0.104505 | -2.48211 | 1 |
| PACSIN2 | 0.0223684 | 0.986062 | -1.60283 | 0.0595036 | 0.997755 | -1.44193 | 0.00077622 | 0.0861162 | -2.33455 | 1 |
| TMEM132A | 0.0229945 | 0.986062 | 1.8069 | 0.0620803 | 0.997755 | 1.58257 | 0.0116388 | 0.164814 | 2.06184 | 2 |
| TIFA | 0.0237892 | 0.986062 | 2.01812 | 0.140777 | 0.997755 | 1.50635 | 0.00945974 | 0.154789 | 2.21388 | 2 |
| NFIC | 0.0241047 | 0.986062 | -1.60059 | 0.0643815 | 0.997755 | -1.44686 | 0.00245852 | 0.114633 | -2.05682 | 1 |
| ITPRIP | 0.0251921 | 0.986062 | -1.32947 | 0.055834 | 0.997755 | -1.25505 | 0.00022723 | 0.0535789 | -1.92064 | 1 |
| UNC93B1 | 0.0256605 | 0.986062 | -1.39857 | 0.0056425 | 0.997755 | -1.57502 | 0.00038501 | 0.0684099 | -1.99626 | 1 |
| PTPRS | 0.0264893 | 0.986062 | 1.7092 | 0.0207479 | 0.997755 | 1.84225 | 0.00501822 | 0.136683 | 2.16854 | 2 |
| C1RL | 0.0278724 | 0.986062 | 1.88482 | 0.0325984 | 0.997755 | 1.75714 | 0.0134421 | 0.172904 | 2.05447 | 2 |
| DHX37 | 0.0288761 | 0.986062 | 2.9574 | 0.809034 | 0.997755 | 1.08531 | 0.00619387 | 0.143075 | 3.58873 | 2 |
| SLC4A11 | 0.0295196 | 0.986062 | -2.03163 | 0.0864793 | 0.997755 | -1.68827 | 0.0451404 | 0.270921 | -1.99191 | 2 |
| MED15 | 0.0300223 | 0.986062 | -1.50582 | 0.132835 | 0.997755 | -1.32106 | 0.00314141 | 0.124854 | -1.99341 | 1 |
| PSMF1 | 0.0309792 | 0.986062 | -1.41933 | 0.0391977 | 0.997755 | -1.39594 | 0.00024437 | 0.054276 | -2.2876 | 1 |
| FER | 0.0329232 | 0.986062 | 1.73856 | 0.0434481 | 0.997755 | 1.68777 | 0.0146881 | 0.176216 | 2.0407 | 1 |
| SLC49A4 | 0.0340397 | 0.986062 | 1.65561 | 0.523119 | 0.997755 | 1.2 | 0.00925551 | 0.154047 | 1.9985 | 1 |
| DTYMK | 0.0345964 | 0.986062 | -1.38914 | 0.0145822 | 0.997755 | -1.49071 | 9.54E-05 | 0.0323208 | -2.45646 | 1 |
| ATP8A2 | 0.0349129 | 0.986062 | 1.79393 | 0.0463628 | 0.997755 | 1.77998 | 0.0163943 | 0.183663 | 1.98512 | 1 |
| H3C2 | 0.0357127 | 0.986062 | -1.87433 | 0.109631 | 0.997755 | -1.59729 | 0.0102684 | 0.158642 | -2.20739 | 2 |
| ZBTB38 | 0.036446 | 0.986062 | 1.93558 | 0.03742 | 0.997755 | 1.97495 | 0.0312408 | 0.236506 | 1.91799 | 3 |
| PPIP5K1 | 0.0367305 | 0.986062 | 1.36932 | 0.0248285 | 0.997755 | 1.38766 | 0.00071757 | 0.0861162 | 1.88895 | 1 |
| H4C14 | 0.036746 | 0.986062 | 1.63128 | 0.127589 | 0.997755 | 1.46576 | 0.00416905 | 0.130631 | 2.1719 | 1 |
| CDC37 | 0.0368494 | 0.986062 | -1.23264 | 0.230155 | 0.997755 | -1.11491 | 0.00010264 | 0.0323208 | -1.818 | 1 |
| F3 | 0.0370313 | 0.986062 | 1.74339 | 0.631268 | 0.997755 | 1.10543 | 0.00346884 | 0.129825 | 2.3851 | 1 |
| EIF4HP1 | 0.0371724 | 0.986062 | -1.57207 | 0.119249 | 0.997755 | -1.39204 | 0.00507979 | 0.136683 | -2.042 | 1 |
| TEX45 | 0.0373448 | 0.986062 | 4.01122 | 0.0462838 | 0.997755 | 2.98802 | 0.038404 | 0.254985 | 3.26151 | 3 |
| RN7SL711P | 0.03877 | 0.986062 | 1.49451 | 0.0523213 | 0.997755 | 1.44104 | 0.00134791 | 0.103851 | 2.20503 | 1 |
| FSCN3 | 0.0393651 | 0.986062 | 1.54856 | 0.064777 | 0.997755 | 1.42013 | 0.00264132 | 0.116303 | 2.05612 | 1 |
| DHCR24 | 0.0402821 | 0.986062 | -1.55379 | 0.0701123 | 0.997755 | -1.46394 | 0.00160692 | 0.109536 | -2.24855 | 1 |
| EGR1 | 0.042037 | 0.986062 | 1.92262 | 0.356182 | 0.997755 | -1.17165 | 0.00240792 | 0.114633 | 3.20632 | 2 |
| H2BC4 | 0.0422675 | 0.986062 | -1.60236 | 0.0348157 | 0.997755 | -1.6517 | 0.00040533 | 0.0686966 | -3.08226 | 1 |
| FIS1 | 0.0425613 | 0.986062 | -1.35027 | 0.649534 | 0.997755 | -1.05574 | 0.00076 | 0.0861162 | -1.89713 | 1 |
| CLPTM1L | 0.0428929 | 0.986062 | -1.30716 | 0.0645899 | 0.997755 | -1.27544 | 0.00080967 | 0.0861162 | -1.82136 | 1 |
| DOK3 | 0.0429079 | 0.986062 | -1.28384 | 0.0195446 | 0.997755 | -1.35206 | 3.29E-05 | 0.0165448 | -2.36152 | 1 |
| DCAKD | 0.0431275 | 0.986062 | -1.801 | 0.37784 | 0.997755 | -1.31344 | 0.0108208 | 0.160845 | -2.40912 | 2 |
| PTGER2 | 0.044151 | 0.986062 | 1.39478 | 0.120467 | 0.997755 | 1.29407 | 0.00301833 | 0.123028 | 1.81071 | 1 |
| PKN1 | 0.0450914 | 0.986062 | -1.41759 | 0.217327 | 0.997755 | -1.22976 | 0.00099815 | 0.0931947 | -2.23686 | 1 |
| MUC2 | 0.0458321 | 0.986062 | 1.41495 | 0.601148 | 0.997755 | 1.09841 | 0.00126123 | 0.101767 | 2.03984 | 1 |
| MED27 | 0.0462169 | 0.986062 | -2.24735 | 0.307679 | 0.997755 | -1.57713 | 0.0314382 | 0.236943 | -2.45618 | 2 |
| SSX3 | 0.0495534 | 0.986062 | 1.5137 | 0.28246 | 0.997755 | 1.25667 | 0.00330601 | 0.127212 | 2.14122 | 1 |
| MEMO1P2 | 0.0512525 | 0.986062 | -1.52423 | 0.36396 | 0.997755 | -1.22433 | 0.0190045 | 0.193153 | -1.82934 | 1 |
| SLC43A1 | 0.0515252 | 0.986062 | -1.41558 | 0.220891 | 0.997755 | -1.23015 | 0.00120204 | 0.100858 | -2.13403 | 1 |
| C1QC | 0.0518964 | 0.986062 | 1.90116 | 0.0234809 | 0.997755 | 2.02673 | 0.0290685 | 0.229939 | 1.98286 | 2 |
| GRK6 | 0.0519896 | 0.986062 | -1.46405 | 0.624127 | 0.997755 | -1.09941 | 0.00197233 | 0.114633 | -2.11305 | 1 |
| KLHL35 | 0.0523438 | 0.986062 | 1.45541 | 0.184527 | 0.997755 | 1.30754 | 0.00311776 | 0.124755 | 2.02379 | 1 |
| FGF8 | 0.054111 | 0.986062 | 1.70333 | 0.225791 | 0.997755 | 1.38785 | 0.0160497 | 0.18112 | 2.00136 | 1 |
| PIGM | 0.0546125 | 0.986062 | -1.5174 | 0.11829 | 0.997755 | -1.38459 | 0.0103751 | 0.158642 | -1.82706 | 1 |
| ADAP1 | 0.0563972 | 0.986062 | -1.5685 | 0.222189 | 0.997755 | -1.32933 | 0.0141006 | 0.17487 | -1.85775 | 1 |
| GBP3 | 0.057739 | 0.986062 | 1.43644 | 0.237534 | 0.997755 | 1.227 | 0.00900942 | 0.15318 | 1.82576 | 1 |
| EIF4H | 0.0591723 | 0.986062 | -1.37967 | 0.20111 | 0.997755 | -1.23412 | 0.0020107 | 0.114633 | -1.93034 | 1 |
| RAC2 | 0.0600737 | 0.986062 | -1.52404 | 0.15036 | 0.997755 | -1.40785 | 0.00432196 | 0.131073 | -2.32537 | 1 |
| MYBL2 | 0.0624305 | 0.986062 | -1.49725 | 0.355627 | 0.997755 | -1.2168 | 0.00541416 | 0.137876 | -1.97134 | 1 |
| SLA | 0.0631835 | 0.986062 | -1.38676 | 0.105285 | 0.997755 | -1.32623 | 0.00331691 | 0.127212 | -1.89191 | 1 |
| BCDIN3D | 0.0641799 | 0.986062 | 1.61991 | 0.0774674 | 0.997755 | 1.52239 | 0.0104666 | 0.159512 | 1.93875 | 1 |
| TRPM2 | 0.0642725 | 0.986062 | -1.54168 | 0.275969 | 0.997755 | -1.22157 | 0.0109874 | 0.16158 | -1.86552 | 1 |
| MGAT4B | 0.0643985 | 0.986062 | -1.27199 | 0.519204 | 0.997755 | -1.0663 | 0.00015475 | 0.0402951 | -2.17568 | 1 |
| Y_RNA | 0.903853 | 0.993835 | -1.03357 | 0.444143 | 0.997755 | -1.12057 | 0.870245 | 0.95135 | -1.03951 | 2 |
| L1CAM | 0.0658284 | 0.986062 | 1.71292 | 0.181503 | 0.997755 | 1.39482 | 0.0350395 | 0.245186 | 1.86912 | 1 |
| PLEKHA3 | 0.0663762 | 0.986062 | 1.47228 | 0.1175 | 0.997755 | 1.38574 | 0.0132314 | 0.17175 | 1.85528 | 1 |
| TMEM109 | 0.0671841 | 0.986062 | -1.51586 | 0.0745786 | 0.997755 | -1.49611 | 0.0187992 | 0.192067 | -1.81451 | 1 |
| CHPF2 | 0.0673121 | 0.986062 | -1.39566 | 0.160356 | 0.997755 | -1.28718 | 0.00042372 | 0.0686966 | -2.38337 | 1 |
| TDRD3 | 0.067517 | 0.986062 | 1.51367 | 0.344605 | 0.997755 | 1.20447 | 0.00589545 | 0.141886 | 2.01314 | 1 |
| GLUL | 0.0687091 | 0.986062 | -1.48427 | 0.310874 | 0.997755 | -1.22975 | 0.00247751 | 0.114633 | -2.16529 | 1 |
| RASA3 | 0.0701081 | 0.986062 | -1.58074 | 0.156692 | 0.997755 | -1.37742 | 0.00613605 | 0.142829 | -2.11175 | 1 |
| SCAMP2 | 0.0715289 | 0.986062 | -1.43989 | 0.109518 | 0.997755 | -1.38357 | 0.00144403 | 0.104505 | -2.2624 | 1 |
| POLDIP3 | 0.0725408 | 0.986062 | -1.27821 | 0.0953168 | 0.997755 | -1.24192 | 0.00058443 | 0.0776694 | -1.92433 | 1 |
| H1-4 | 0.0732655 | 0.986062 | -1.58695 | 0.155788 | 0.997755 | -1.41549 | 0.00794618 | 0.15011 | -2.18724 | 1 |
| POC1B-GALNT4 | 0.0740282 | 0.986062 | 1.77612 | 0.0869411 | 0.997755 | 1.69467 | 0.0441333 | 0.268878 | 1.93692 | 1 |
| DYRK3 | 0.0742455 | 0.986062 | 1.62531 | 0.186247 | 0.997755 | 1.41508 | 0.0254641 | 0.21611 | 1.81152 | 1 |
| PHB1 | 0.0746155 | 0.986062 | -1.45474 | 0.343188 | 0.997755 | -1.21995 | 0.0062808 | 0.143075 | -1.98761 | 1 |
| NRXN3 | 0.0753445 | 0.986062 | 1.35415 | 0.475681 | 0.997755 | 1.143 | 0.00302214 | 0.123028 | 1.89352 | 1 |
| AP2M1 | 0.0767035 | 0.986062 | -1.27694 | 0.098908 | 0.997755 | -1.24229 | 0.00058257 | 0.0776694 | -1.89853 | 1 |
| BCAP31 | 0.0784733 | 0.986062 | -1.7028 | 0.590731 | 0.997755 | -1.17941 | 0.0010462 | 0.0931947 | -4.04852 | 1 |
| OTX2 | 0.0785369 | 0.986062 | 1.57013 | 0.101673 | 0.997755 | 1.49273 | 0.0346166 | 0.244731 | 1.82327 | 1 |
| H2BP6 | 0.0808943 | 0.986062 | -1.41279 | 0.064583 | 0.997755 | -1.49254 | 0.00126289 | 0.101767 | -2.40738 | 1 |
| SLC7A5 | 0.0810698 | 0.986062 | -1.44133 | 0.165443 | 0.997755 | -1.32668 | 0.00235009 | 0.114633 | -2.22636 | 1 |
| CCDC88B | 0.0812261 | 0.986062 | 1.51726 | 0.0447937 | 0.997755 | 1.55774 | 0.00106663 | 0.0931947 | 2.58201 | 1 |
| STX11 | 0.082228 | 0.986062 | 1.43978 | 0.260148 | 0.997755 | 1.23347 | 0.00411947 | 0.130631 | 2.03777 | 1 |
| SLC26A6 | 0.0835856 | 0.986062 | 1.30967 | 0.00239791 | 0.992848 | 1.7835 | 0.00056747 | 0.0776694 | 2.05829 | 1 |
| ZNF211 | 0.0841271 | 0.986062 | 1.76499 | 0.339023 | 0.997755 | 1.2945 | 0.0462152 | 0.273506 | 1.98158 | 1 |
| LRPAP1 | 0.0844542 | 0.986062 | -1.66257 | 0.244367 | 0.997755 | -1.42428 | 0.0107663 | 0.160845 | -2.32028 | 1 |
| ZNF664 | 0.0850558 | 0.986062 | 1.49708 | 0.019646 | 0.997755 | 1.69212 | 0.00551693 | 0.139102 | 1.98113 | 1 |
| PPP6R1 | 0.0852962 | 0.986062 | -1.73347 | 0.288075 | 0.997755 | -1.30249 | 0.0134518 | 0.172904 | -2.28582 | 1 |
| MOSPD3 | 0.0854256 | 0.986062 | -1.56794 | 0.191154 | 0.997755 | -1.44013 | 0.0132185 | 0.17175 | -2.11964 | 1 |
| FOXR1 | 0.086865 | 0.986062 | 1.50214 | 0.055915 | 0.997755 | 1.66774 | 0.00451284 | 0.133381 | 2.28799 | 1 |
| JUNB | 0.0870773 | 0.986062 | 1.46811 | 0.223595 | 0.997755 | 1.2779 | 0.0106905 | 0.160496 | 1.99248 | 1 |
| NOL12 | 0.0877208 | 0.986062 | -1.46938 | 0.243621 | 0.997755 | -1.25079 | 0.00207609 | 0.114633 | -2.36217 | 1 |
| DEDD | 0.088383 | 0.986062 | -1.32267 | 0.282981 | 0.997755 | -1.19444 | 0.00176901 | 0.111863 | -1.95761 | 1 |
| CCDC146 | 0.0893048 | 0.986062 | 1.52731 | 0.0820079 | 0.997755 | 1.53979 | 0.00612415 | 0.142829 | 2.1501 | 1 |
| MTERF4 | 0.0898618 | 0.986062 | 1.3476 | 0.11021 | 0.997755 | 1.29359 | 0.00304022 | 0.123255 | 1.85501 | 1 |
| LGALS9 | 0.0898688 | 0.986062 | -1.67507 | 0.24738 | 0.997755 | -1.27018 | 0.0207436 | 0.199549 | -2.13791 | 1 |
| SDF2L1 | 0.0901126 | 0.986062 | 1.68804 | 0.0786724 | 0.997755 | 1.63893 | 0.0248122 | 0.212317 | 1.95209 | 1 |
| LSAMP | 0.0906372 | 0.986062 | 1.98723 | 0.6476 | 0.997755 | 1.196 | 0.0493157 | 0.281487 | 2.17847 | 1 |
| CYREN | 0.0908393 | 0.986062 | -1.28626 | 0.508416 | 0.997755 | -1.10283 | 0.00216483 | 0.114633 | -1.86038 | 1 |
| RAD52 | 0.0918333 | 0.986062 | -1.48832 | 0.342747 | 0.997755 | -1.26524 | 0.00855598 | 0.150763 | -2.12032 | 1 |
| NRG4 | 0.0923153 | 0.986062 | 1.64508 | 0.257988 | 0.997755 | 1.44419 | 0.0158629 | 0.180105 | 2.2973 | 1 |
| DPF2 | 0.0942275 | 0.986062 | -1.46805 | 0.132855 | 0.997755 | -1.42648 | 0.00206206 | 0.114633 | -2.4528 | 1 |
| NIPSNAP1 | 0.0945293 | 0.986062 | -1.34065 | 0.127611 | 0.997755 | -1.27678 | 0.00084202 | 0.0877033 | -2.15919 | 1 |
| ATP6V0D1 | 0.0978694 | 0.986062 | -1.37758 | 0.524741 | 0.997755 | -1.11608 | 0.00234761 | 0.114633 | -2.04098 | 1 |
| MYADM | 0.0995357 | 0.986062 | -1.44999 | 0.0705752 | 0.997755 | -1.51081 | 0.00473377 | 0.136077 | -2.21673 | 1 |
| KRI1 | 0.100565 | 0.986062 | -1.27453 | 0.293576 | 0.997755 | -1.15099 | 0.00137998 | 0.103851 | -1.83682 | 1 |
| FASTK | 0.101381 | 0.986062 | 1.52462 | 0.598221 | 0.997755 | 1.12896 | 0.0337967 | 0.243179 | 1.92218 | 1 |
| OPTC | 0.10242 | 0.986062 | 1.62582 | 0.616362 | 0.997755 | 1.1059 | 0.0140055 | 0.17487 | 2.17922 | 1 |
| H2AC11 | 0.102429 | 0.986062 | -1.94935 | 0.205207 | 0.997755 | -1.66287 | 0.0218062 | 0.201908 | -2.65806 | 1 |
| NAPEPLD | 0.102519 | 0.986062 | 1.55221 | 0.30171 | 0.997755 | 1.28453 | 0.0101546 | 0.158271 | 2.14129 | 1 |
| TAF8 | 0.107028 | 0.986062 | 1.56154 | 0.525167 | 0.997755 | 1.15176 | 0.0287218 | 0.22879 | 1.93119 | 1 |
| EXOSC5 | 0.107607 | 0.986062 | -1.3813 | 0.507274 | 0.997755 | -1.10391 | 0.00905018 | 0.15318 | -1.92443 | 1 |
| YIF1A | 0.108211 | 0.986062 | -1.61304 | 0.385046 | 0.997755 | -1.29091 | 0.0288949 | 0.229684 | -1.89761 | 1 |
| SNX17 | 0.108575 | 0.986062 | -1.5229 | 0.248448 | 0.997755 | -1.33525 | 0.0199994 | 0.196545 | -1.90052 | 1 |
| CORO7-PAM16 | 0.109068 | 0.986062 | -1.30026 | 0.0515478 | 0.997755 | -1.43217 | 0.00179472 | 0.111863 | -2.078 | 1 |
| BLOC1S1 | 0.110765 | 0.986062 | -1.1646 | 0.0448645 | 0.997755 | -1.21528 | 7.47E-05 | 0.0297022 | -1.87435 | 1 |
| SHMT2 | 0.11105 | 0.986062 | -1.28715 | 0.12633 | 0.997755 | -1.284 | 0.00136335 | 0.103851 | -2.04643 | 1 |
| TRMT61A | 0.111712 | 0.986062 | 1.51773 | 0.840248 | 0.997755 | 1.02915 | 0.0144371 | 0.175417 | 2.12977 | 1 |
| H2AC19 | 0.113467 | 0.986062 | -1.38229 | 0.201145 | 0.997755 | -1.29785 | 0.00372014 | 0.130631 | -2.04069 | 1 |
| FXYD2 | 0.113615 | 0.986062 | 1.29785 | 0.0311732 | 0.997755 | 1.48135 | 0.00278677 | 0.118894 | 1.88515 | 1 |
| AGAP3 | 0.114112 | 0.986062 | 1.36216 | 0.491959 | 0.997755 | 1.13109 | 0.0103649 | 0.158642 | 1.85088 | 1 |
| PRMT2 | 0.114143 | 0.986062 | -1.33544 | 0.36679 | 0.997755 | -1.14589 | 0.00836522 | 0.150763 | -1.81073 | 1 |
| ATF6B | 0.114832 | 0.986062 | -1.54519 | 0.310332 | 0.997755 | -1.29485 | 0.00817972 | 0.150763 | -2.62885 | 1 |
| RALY | 0.115197 | 0.986062 | -1.4427 | 0.255866 | 0.997755 | -1.31453 | 0.00463508 | 0.136077 | -2.31539 | 1 |
| SH3BGRL3 | 0.115696 | 0.986062 | -1.28888 | 0.0609428 | 0.997755 | -1.38008 | 0.00236906 | 0.114633 | -1.92921 | 1 |
| NDST1 | 0.115831 | 0.986062 | -1.49903 | 0.191561 | 0.997755 | -1.37208 | 0.00759051 | 0.148279 | -2.18546 | 1 |
| EPN1 | 0.115943 | 0.986062 | 1.59588 | 0.0761945 | 0.997755 | 1.58805 | 0.0135627 | 0.173097 | 2.03233 | 1 |
| DRAP1 | 0.116322 | 0.986062 | -1.41456 | 0.0998581 | 0.997755 | -1.38273 | 0.00095741 | 0.0923534 | -2.68979 | 1 |
| LINC00656 | 0.118069 | 0.986062 | -1.47152 | 0.328975 | 0.997755 | -1.30561 | 0.00377461 | 0.130631 | -2.65489 | 1 |
| H4C5 | 0.118226 | 0.986062 | -1.86204 | 0.236226 | 0.997755 | -1.59629 | 0.0102836 | 0.158642 | -2.87048 | 1 |
| COL11A1 | 0.119017 | 0.986062 | 1.66947 | 0.0194857 | 0.997755 | 2.28498 | 0.0315881 | 0.236943 | 2.29324 | 2 |
| SMARCD3 | 0.119602 | 0.986062 | 1.46162 | 0.117712 | 0.997755 | 1.4753 | 0.0193673 | 0.194258 | 1.95814 | 1 |
| ZNF428 | 0.119935 | 0.986062 | 1.66827 | 0.063392 | 0.997755 | 1.7274 | 0.0130336 | 0.170933 | 2.21985 | 1 |
| HS3ST1 | 0.120256 | 0.986062 | 1.60926 | 0.497263 | 0.997755 | 1.26147 | 0.0283928 | 0.227852 | 2.10467 | 1 |
| PGD | 0.121076 | 0.986062 | -1.31025 | 0.336647 | 0.997755 | -1.18187 | 0.00182944 | 0.111863 | -2.03203 | 1 |
| SDC2 | 0.12215 | 0.986062 | 1.29178 | 0.0315787 | 0.997755 | 1.42854 | 0.00132462 | 0.103851 | 1.94306 | 1 |
| IPO4 | 0.122224 | 0.986062 | -1.39321 | 0.235864 | 0.997755 | -1.29564 | 0.00244621 | 0.114633 | -2.2773 | 1 |
| LMAN2 | 0.125463 | 0.986062 | -1.62084 | 0.389088 | 0.997755 | -1.32828 | 0.00567689 | 0.140785 | -3.0207 | 1 |
| RNF214 | 0.131682 | 0.986062 | 1.34443 | 0.13348 | 0.997755 | 1.30699 | 0.00582888 | 0.141886 | 1.86545 | 1 |
| CLPP | 0.132868 | 0.986062 | -1.25462 | 0.167027 | 0.997755 | -1.24892 | 0.00212664 | 0.114633 | -1.88999 | 1 |
| DNAJC17 | 0.133176 | 0.986062 | 1.51509 | 0.919273 | 0.997755 | 1.04701 | 0.035066 | 0.245186 | 1.85339 | 1 |
| SIVA1 | 0.133276 | 0.986062 | -1.54481 | 0.0701871 | 0.997755 | -1.56218 | 0.0065074 | 0.143764 | -2.53724 | 1 |
| MIR1282 | 0.136444 | 0.986062 | -1.51185 | 0.260393 | 0.997755 | -1.35385 | 0.00234662 | 0.114633 | -2.87354 | 1 |
| FN3KRP | 0.137253 | 0.986062 | -1.43898 | 0.6454 | 0.997755 | 1.12537 | 0.0188121 | 0.192067 | -1.94916 | 1 |
| ADGRG1 | 0.138655 | 0.986062 | 1.41016 | 0.352591 | 0.997755 | 1.28978 | 0.0133283 | 0.172049 | 1.95016 | 1 |
| ALG3 | 0.139952 | 0.986062 | -1.25 | 0.0547583 | 0.997755 | -1.34553 | 0.00189715 | 0.112939 | -1.81101 | 1 |
| MSRB1 | 0.14053 | 0.986062 | -1.2952 | 0.230452 | 0.997755 | -1.25091 | 0.00251637 | 0.114633 | -2.05724 | 1 |
| H2AC20 | 0.141114 | 0.986062 | -1.54815 | 0.114145 | 0.997755 | -1.51332 | 0.0281051 | 0.226869 | -2.01717 | 1 |
| PLEKHM2 | 0.142627 | 0.986062 | -1.39686 | 0.313477 | 0.997755 | -1.23777 | 0.00324506 | 0.126679 | -2.28921 | 1 |
| RNU6-622P | 0.143608 | 0.986062 | 1.94272 | 0.311463 | 0.997755 | 1.38739 | 0.0482809 | 0.279061 | 2.48117 | 1 |
| ACTBP6 | 0.144856 | 0.986062 | -1.42351 | 0.267771 | 0.997755 | -1.33414 | 0.0116755 | 0.164953 | -2.20621 | 1 |
| LSP1 | 0.145387 | 0.986062 | -1.37332 | 0.551273 | 0.997755 | -1.13333 | 0.0060478 | 0.142829 | -1.96226 | 1 |
| NHP2 | 0.145695 | 0.986062 | -1.51659 | 0.342459 | 0.997755 | -1.31104 | 0.00654243 | 0.143764 | -2.60059 | 1 |
| H2BP1 | 0.207347 | 0.986062 | -1.60708 | 0.312525 | 0.997755 | -1.57846 | 0.0227868 | 0.205221 | -2.91287 | 2 |
| ACVR2A | 0.147121 | 0.986062 | 1.35718 | 0.034662 | 0.997755 | 1.5476 | 0.00888119 | 0.152168 | 1.8167 | 1 |
| TEPP | 0.147721 | 0.986062 | 1.56075 | 0.114142 | 0.997755 | 1.69143 | 0.00427887 | 0.130631 | 3.01556 | 1 |
| USP44 | 0.149042 | 0.986062 | 1.43716 | 0.175673 | 0.997755 | 1.45664 | 0.0232289 | 0.207222 | 1.93065 | 1 |
| H3C11 | 0.149695 | 0.986062 | -1.48202 | 0.328822 | 0.997755 | -1.3234 | 0.0224633 | 0.204993 | -1.9588 | 1 |
| VPS25 | 0.151168 | 0.986062 | -1.48937 | 0.565287 | 0.997755 | -1.20651 | 0.00793664 | 0.15011 | -2.9915 | 1 |
| UBL3 | 0.151594 | 0.986062 | 1.31516 | 0.188052 | 0.997755 | 1.29498 | 0.00815559 | 0.150763 | 1.87988 | 1 |
| EMP3 | 0.153149 | 0.986062 | -1.28777 | 0.258085 | 0.997755 | -1.21319 | 0.00297276 | 0.121674 | -1.98108 | 1 |
| H2BP3 | 0.154071 | 0.986062 | -1.28387 | 0.115751 | 0.997755 | -1.32259 | 0.00179573 | 0.111863 | -2.00475 | 1 |
| ELOF1 | 0.154615 | 0.986062 | -2.07553 | 0.348342 | 0.997755 | -1.57068 | 0.0327127 | 0.239323 | -2.91793 | 1 |
| PAF1 | 0.163446 | 0.986062 | -1.40812 | 0.709191 | 0.997755 | -1.08895 | 0.0143885 | 0.175417 | -1.91193 | 1 |
| TMEM169 | 0.163507 | 0.986062 | 1.53018 | 0.319902 | 0.997755 | 1.43786 | 0.0257916 | 0.21798 | 2.28114 | 1 |
| RCAN1 | 0.163731 | 0.986062 | 1.30589 | 0.901944 | 0.997755 | -1.01079 | 0.00400904 | 0.130631 | 1.91288 | 1 |
| ZC3H6 | 0.165924 | 0.986062 | 1.60489 | 0.617901 | 0.997755 | 1.14765 | 0.0227468 | 0.205221 | 1.9941 | 1 |
| NFE2 | 0.166175 | 0.986062 | -1.45017 | 0.236012 | 0.997755 | -1.39407 | 0.0272806 | 0.223788 | -1.87122 | 1 |
| CCL5 | 0.166386 | 0.986062 | -1.32452 | 0.0518369 | 0.997755 | -1.51018 | 0.00374834 | 0.130631 | -2.04808 | 1 |
| BCLAF3 | 0.166552 | 0.986062 | 1.36775 | 0.0863236 | 0.997755 | 1.59803 | 0.0169214 | 0.184923 | 1.85275 | 1 |
| MIF | 0.166938 | 0.986062 | -1.67715 | 0.21285 | 0.997755 | -1.50625 | 0.00639924 | 0.143764 | -3.11002 | 1 |
| MIF-AS1 | 0.167338 | 0.986062 | -1.76952 | 0.240517 | 0.997755 | -1.55211 | 0.00611142 | 0.142829 | -3.49034 | 1 |
| CPLANE1 | 0.167728 | 0.986062 | 1.33532 | 0.0864669 | 0.997755 | 1.43791 | 0.00211316 | 0.114633 | 2.2039 | 1 |
| EDC3 | 0.169385 | 0.986062 | -1.54813 | 0.401602 | 0.997755 | -1.29086 | 0.0407894 | 0.260808 | -1.89403 | 1 |
| FARSA | 0.170005 | 0.986062 | -1.2014 | 0.483624 | 0.997755 | -1.08823 | 0.00052726 | 0.074423 | -1.9673 | 1 |
| SARS1 | 0.170319 | 0.986062 | -1.32998 | 0.736071 | 0.997755 | -1.07863 | 0.0115135 | 0.164355 | -1.92414 | 1 |
| MRPS18A | 0.170891 | 0.986062 | -1.68896 | 0.280046 | 0.997755 | -1.49346 | 0.0428019 | 0.266694 | -2.23185 | 1 |
| GCC1 | 0.171677 | 0.986062 | 1.33658 | 0.0875863 | 0.997755 | 1.42233 | 0.0113497 | 0.163844 | 1.81816 | 1 |
| PRDX2 | 0.171885 | 0.986062 | -1.49105 | 0.146617 | 0.997755 | -1.53109 | 0.00511638 | 0.136683 | -2.68166 | 1 |
| MACC1 | 0.172377 | 0.986062 | 1.65432 | 0.496465 | 0.997755 | 1.22669 | 0.0245341 | 0.211254 | 2.43937 | 1 |
| DSTYK | 0.173341 | 0.986062 | 1.46299 | 0.981713 | 0.999836 | 1.03701 | 0.00694598 | 0.143764 | 2.5828 | 1 |
| ARF1 | 0.173584 | 0.986062 | -1.48453 | 0.366596 | 0.997755 | -1.30269 | 0.00728451 | 0.1452 | -2.41759 | 1 |
| EIF4EBP1 | 0.174162 | 0.986062 | -1.24291 | 0.0280785 | 0.997755 | -1.46876 | 0.00071738 | 0.0861162 | -2.10033 | 1 |
| ZBTB41 | 0.17679 | 0.986062 | 1.27462 | 0.116317 | 0.997755 | 1.33516 | 0.00818247 | 0.150763 | 1.82525 | 1 |
| LITAF | 0.177137 | 0.986062 | -1.26327 | 0.0702543 | 0.997755 | -1.42065 | 0.00125281 | 0.101767 | -2.23874 | 1 |
| GAL3ST4 | 0.17759 | 0.986062 | 1.38177 | 0.124741 | 0.997755 | 1.41062 | 0.014722 | 0.176216 | 1.87681 | 1 |
| H2AC16 | 0.178513 | 0.986062 | -2.46776 | 0.307735 | 0.997755 | -2.11203 | 0.00983457 | 0.156802 | -6.40331 | 1 |
| FBXO44 | 0.178891 | 0.986062 | 1.52881 | 0.303527 | 0.997755 | 1.35571 | 0.0139122 | 0.17487 | 2.50166 | 1 |
| YARS2 | 0.182983 | 0.986062 | 1.45536 | 0.209959 | 0.997755 | 1.47043 | 0.0148279 | 0.176231 | 2.3378 | 1 |
| PAM16 | 0.183503 | 0.986062 | -1.33057 | 0.097712 | 0.997755 | -1.50762 | 0.00239993 | 0.114633 | -2.60318 | 1 |
| CSNK1G2 | 0.183691 | 0.986062 | -1.41105 | 0.148613 | 0.997755 | -1.50898 | 0.00201813 | 0.114633 | -2.92053 | 1 |
| RUNX1T1 | 0.183911 | 0.986062 | 1.48669 | 0.441014 | 0.997755 | 1.23614 | 0.0288945 | 0.229684 | 2.19137 | 1 |
| MRPL4 | 0.184995 | 0.986062 | -1.26185 | 0.547939 | 0.997755 | -1.08328 | 0.00273168 | 0.117206 | -2.08603 | 1 |
| EIF3G | 0.186289 | 0.986062 | -1.27808 | 0.975872 | 0.998599 | -1.00874 | 0.00664046 | 0.143764 | -1.81407 | 1 |
| NHP2P2 | 0.187119 | 0.986062 | -1.74572 | 0.401074 | 0.997755 | -1.44856 | 0.0131676 | 0.171678 | -3.17006 | 1 |
| ANAPC11 | 0.187433 | 0.986062 | -1.39274 | 0.26829 | 0.997755 | -1.32615 | 0.0035937 | 0.130631 | -2.43854 | 1 |
| THEM6 | 0.187615 | 0.986062 | 1.40574 | 0.881228 | 0.997755 | 1.03914 | 0.0314724 | 0.236943 | 1.93805 | 1 |
| TXN2 | 0.188077 | 0.986062 | -1.3613 | 0.266884 | 0.997755 | -1.3026 | 0.00498694 | 0.136683 | -2.3511 | 1 |
| RPL32P36 | 0.188169 | 0.986062 | 1.35854 | 0.280111 | 0.997755 | 1.29933 | 0.00777136 | 0.149729 | 2.08196 | 1 |
| SMARCB1 | 0.1885 | 0.986062 | -1.26717 | 0.620811 | 0.997755 | -1.07551 | 0.00339771 | 0.129186 | -1.89421 | 1 |
| PRMT1 | 0.189178 | 0.986062 | -1.38679 | 0.237006 | 0.997755 | -1.36706 | 0.00626323 | 0.143075 | -2.36829 | 1 |
| TIMM50 | 0.189466 | 0.986062 | -1.30391 | 0.491959 | 0.997755 | -1.14472 | 0.00481092 | 0.136077 | -1.9787 | 1 |
| ACBD6 | 0.190394 | 0.986062 | 1.36402 | 0.0813742 | 0.997755 | 1.46544 | 0.0166001 | 0.184299 | 1.82534 | 1 |
| ADORA3 | 0.190651 | 0.986062 | -1.22181 | 0.0468737 | 0.997755 | -1.37939 | 0.00043744 | 0.0686966 | -2.22387 | 1 |
| P4HB | 0.19206 | 0.986062 | -1.2627 | 0.281812 | 0.997755 | -1.21234 | 0.00656979 | 0.143764 | -1.81534 | 1 |
| UBE2L6 | 0.19352 | 0.986062 | -1.547 | 0.455184 | 0.997755 | -1.22279 | 0.0198222 | 0.196054 | -2.22467 | 1 |
| CAMSAP2 | 0.193742 | 0.986062 | 1.41531 | 0.578902 | 0.997755 | 1.14038 | 0.00689462 | 0.143764 | 2.15452 | 1 |
| PTAFR | 0.194616 | 0.986062 | -1.22951 | 0.118633 | 0.997755 | -1.30589 | 0.00323927 | 0.126679 | -1.82058 | 1 |
| ANKRD18A | 0.194747 | 0.986062 | 1.57622 | 0.220834 | 0.997755 | 1.50657 | 0.0317475 | 0.236943 | 2.03963 | 1 |
| COLGALT1 | 0.195158 | 0.986062 | -1.24264 | 0.404364 | 0.997755 | -1.1563 | 0.00238328 | 0.114633 | -1.91995 | 1 |
| CHID1 | 0.196008 | 0.986062 | -1.16068 | 0.084917 | 0.997755 | -1.22889 | 0.00027083 | 0.0579221 | -1.87402 | 1 |
| ECH1 | 0.196098 | 0.986062 | -1.62028 | 0.515861 | 0.997755 | -1.2932 | 0.0125636 | 0.168752 | -2.98629 | 1 |
| ADAT1 | 0.196899 | 0.986062 | -1.37674 | 0.150534 | 0.997755 | -1.4413 | 0.00985308 | 0.156802 | -2.01457 | 1 |
| SCAMP3 | 0.197249 | 0.986062 | -1.32395 | 0.271561 | 0.997755 | -1.2645 | 0.00386586 | 0.130631 | -2.0782 | 1 |
| TREM1 | 0.197455 | 0.986062 | -1.38434 | 0.441136 | 0.997755 | -1.22191 | 0.0166555 | 0.184555 | -1.92118 | 1 |
| ITPA | 0.198515 | 0.986062 | -1.30016 | 0.357887 | 0.997755 | -1.21055 | 0.00272845 | 0.117206 | -2.21584 | 1 |
| ATP5MC2 | 0.199805 | 0.986062 | -1.40414 | 0.361606 | 0.997755 | -1.31563 | 0.00558353 | 0.139909 | -2.75231 | 1 |
| ZFYVE16 | 0.200849 | 0.986062 | 1.30813 | 0.341395 | 0.997755 | 1.21087 | 0.00807329 | 0.150763 | 2.00793 | 1 |
| ELOVL1 | 0.203237 | 0.986062 | -1.62546 | 0.287803 | 0.997755 | -1.4828 | 0.0149432 | 0.176855 | -2.76741 | 1 |
| NQO1-DT | 0.203257 | 0.986062 | -1.3406 | 0.69756 | 0.997755 | -1.09947 | 0.00708169 | 0.144086 | -2.14384 | 1 |
| LINC00667 | 0.204202 | 0.986062 | 1.3991 | 0.112954 | 0.997755 | 1.55851 | 0.0399945 | 0.25891 | 1.83512 | 1 |
| ACTBP4 | 0.2052 | 0.986062 | -1.40573 | 0.17783 | 0.997755 | -1.45696 | 0.00643506 | 0.143764 | -2.46931 | 1 |
| MYRF | 0.206087 | 0.986062 | 1.89566 | 0.301941 | 0.997755 | 1.62591 | 0.0443915 | 0.268878 | 2.53322 | 1 |
| FAM110B | 0.207047 | 0.986062 | 1.387 | 0.704101 | 0.997755 | 1.11578 | 0.0349509 | 0.245176 | 1.93905 | 1 |
| H2BC21 | 0.210438 | 0.986062 | -1.53089 | 0.21455 | 0.997755 | -1.52836 | 0.00986162 | 0.156802 | -2.81559 | 1 |
| RPL29P11 | 0.210806 | 0.986062 | -1.28771 | 0.20624 | 0.997755 | -1.29833 | 0.00678704 | 0.143764 | -2.02635 | 1 |
| LAPTM5 | 0.21299 | 0.986062 | -1.24812 | 0.258998 | 0.997755 | -1.23268 | 0.00399578 | 0.130631 | -1.92467 | 1 |
| SERF2 | 0.217745 | 0.986062 | -1.2704 | 0.184196 | 0.997755 | -1.30354 | 0.00965292 | 0.156425 | -1.82058 | 1 |
| DDRGK1 | 0.221016 | 0.986062 | -1.32388 | 0.608672 | 0.997755 | -1.13216 | 0.0139599 | 0.17487 | -2.0352 | 1 |
| ATP1B1 | 0.224226 | 0.986062 | 1.39992 | 0.0616875 | 0.997755 | 1.69935 | 0.0223623 | 0.204622 | 2.03702 | 1 |
| UCK1 | 0.225608 | 0.986062 | 1.25883 | 0.648151 | 0.997755 | -1.05259 | 0.00396089 | 0.130631 | 2.00319 | 1 |
| NOB1 | 0.227726 | 0.986062 | -1.41577 | 0.414807 | 0.997755 | -1.28132 | 0.021731 | 0.201754 | -2.0048 | 1 |
| PIGU | 0.228095 | 0.986062 | -1.23653 | 0.0943286 | 0.997755 | -1.38103 | 0.00533019 | 0.137855 | -1.86362 | 1 |
| GLE1 | 0.230037 | 0.986062 | 1.3571 | 0.118635 | 0.997755 | 1.51295 | 0.0324134 | 0.238378 | 1.86771 | 1 |
| C11orf65 | 0.232733 | 0.986062 | 1.39642 | 0.621442 | 0.997755 | -1.15035 | 0.0421904 | 0.264509 | 2.07397 | 1 |
| PPP1CA | 0.234199 | 0.986062 | -1.26386 | 0.179435 | 0.997755 | -1.30909 | 0.00419378 | 0.130631 | -1.9769 | 1 |
| GLULP4 | 0.234244 | 0.986062 | -1.30928 | 0.169661 | 0.997755 | -1.35034 | 0.0172038 | 0.186309 | -1.8998 | 1 |
| SLC25A6P2 | 0.238265 | 0.986062 | -1.30009 | 0.449599 | 0.997755 | -1.16537 | 0.00932525 | 0.154253 | -2.1102 | 1 |
| LINC00303 | 0.238416 | 0.986062 | 1.62457 | 0.709064 | 0.997755 | 1.18203 | 0.0326368 | 0.239162 | 2.28987 | 1 |
| RAB11B | 0.240124 | 0.986062 | -1.35159 | 0.296656 | 0.997755 | -1.29494 | 0.00517499 | 0.136683 | -2.25909 | 1 |
| H2BC7 | 0.24054 | 0.986062 | -1.90469 | 0.223122 | 0.997755 | -1.91823 | 0.00782844 | 0.150005 | -4.64928 | 1 |
| DUS1L | 0.240938 | 0.986062 | -1.1727 | 0.163014 | 0.997755 | -1.20733 | 0.00068874 | 0.0861162 | -1.88193 | 1 |
| ENO3 | 0.241977 | 0.986062 | -1.31649 | 0.215157 | 0.997755 | -1.37537 | 0.00245999 | 0.114633 | -2.93045 | 1 |
| PTPN7 | 0.24277 | 0.986062 | -1.52584 | 0.299018 | 0.997755 | -1.41424 | 0.00589424 | 0.141886 | -2.98892 | 1 |
| ATF7IP2 | 0.244036 | 0.986062 | 1.29847 | 0.21242 | 0.997755 | 1.32997 | 0.00570322 | 0.140913 | 2.38292 | 1 |
| SLC17A9 | 0.244933 | 0.986062 | -1.35478 | 0.173505 | 0.997755 | -1.4942 | 0.0151318 | 0.177609 | -2.47825 | 1 |
| RPL10P2 | 0.245718 | 0.986062 | -1.28167 | 0.232882 | 0.997755 | -1.30226 | 0.00838075 | 0.150763 | -2.03295 | 1 |
| C12orf57 | 0.246261 | 0.986062 | -1.6155 | 0.395735 | 0.997755 | -1.37685 | 0.0058134 | 0.141886 | -3.83898 | 1 |
| TOP3B | 0.248184 | 0.986062 | -1.19029 | 0.890559 | 0.997755 | -1.02425 | 0.00139618 | 0.104389 | -1.91144 | 1 |
| CARD19 | 0.252797 | 0.986062 | -1.40809 | 0.501745 | 0.997755 | -1.23301 | 0.0125213 | 0.168752 | -3.12075 | 1 |
| RBM23 | 0.253297 | 0.986062 | -1.42756 | 0.407972 | 0.997755 | -1.287 | 0.0285897 | 0.228355 | -1.86451 | 1 |
| H2BC12 | 0.255627 | 0.986062 | -1.49417 | 0.318365 | 0.997755 | -1.38969 | 0.0159375 | 0.180438 | -2.46509 | 1 |
| NMNAT3 | 0.255751 | 0.986062 | 1.54178 | 0.613992 | 0.997755 | 1.21483 | 0.00812934 | 0.150763 | 4.55338 | 1 |
| DDX43 | 0.257563 | 0.986062 | 1.26447 | 0.353207 | 0.997755 | 1.2026 | 0.0143419 | 0.175417 | 1.85652 | 1 |
| MSTO2P | 0.259016 | 0.986062 | -1.18966 | 0.986254 | 0.999906 | -1.00085 | 0.00101991 | 0.0931947 | -2.0126 | 1 |
| HNRNPA1P11 | 0.259721 | 0.986062 | -1.62723 | 0.672974 | 0.997755 | -1.21884 | 0.0333562 | 0.241907 | -2.51258 | 1 |
| CDCA3 | 0.260436 | 0.986062 | -1.20322 | 0.900413 | 0.997755 | -1.00023 | 0.00921129 | 0.154047 | -1.84537 | 1 |
| TUBB1 | 0.260697 | 0.986062 | -1.5074 | 0.374158 | 0.997755 | -1.42365 | 0.0115909 | 0.164814 | -2.9644 | 1 |
| FAM98B | 0.261311 | 0.986062 | 1.69953 | 0.350658 | 0.997755 | 1.54725 | 0.0148062 | 0.176231 | 5.22271 | 1 |
| ASB8 | 0.261639 | 0.986062 | -1.38562 | 0.577064 | 0.997755 | -1.2101 | 0.0199259 | 0.196207 | -2.2048 | 1 |
| RIC3 | 0.262458 | 0.986062 | 1.3065 | 0.923487 | 0.997755 | 1.07077 | 0.0296179 | 0.2316 | 1.80762 | 1 |
| MIER3 | 0.262792 | 0.986062 | 1.32192 | 0.816666 | 0.997755 | 1.05793 | 0.0216277 | 0.201695 | 1.81303 | 1 |
| PLOD1 | 0.264061 | 0.986062 | -1.22908 | 0.343196 | 0.997755 | -1.16016 | 0.00292061 | 0.120519 | -2.14414 | 1 |
| MIR3655 | 0.264604 | 0.986062 | -1.4385 | 0.191241 | 0.997755 | -1.41829 | 0.0495288 | 0.281679 | -1.92976 | 1 |
| RNF220 | 0.267845 | 0.986062 | -1.35835 | 0.386766 | 0.997755 | -1.27799 | 0.00382074 | 0.130631 | -2.79199 | 1 |
| FAM9B | 0.268037 | 0.986062 | 1.40551 | 0.644724 | 0.997755 | -1.08332 | 0.0496079 | 0.281877 | 1.8604 | 1 |
| BCKDK | 0.270487 | 0.986062 | -1.27774 | 0.405474 | 0.997755 | -1.17173 | 0.0034993 | 0.129994 | -2.27831 | 1 |
| H2BC12L | 0.271073 | 0.986062 | -1.60267 | 0.324376 | 0.997755 | -1.52629 | 0.0225116 | 0.205083 | -2.65454 | 1 |
| SNRPCP2 | 0.271125 | 0.986062 | -1.34027 | 0.315769 | 0.997755 | -1.31054 | 0.00905023 | 0.15318 | -2.16675 | 1 |
| KCNJ15 | 0.271171 | 0.986062 | 1.31846 | 0.0886002 | 0.997755 | 1.52685 | 0.0135814 | 0.173097 | 2.0698 | 1 |
| DIO3OS | 0.271778 | 0.986062 | 1.56838 | 0.65687 | 0.997755 | 1.31867 | 0.040315 | 0.259428 | 2.89105 | 1 |
| NTNG1 | 0.273721 | 0.986062 | 1.39605 | 0.109548 | 0.997755 | 1.51897 | 0.0128181 | 0.169985 | 2.16199 | 1 |
| RNASEK-C17orf49 | 0.277529 | 0.986062 | -1.26596 | 0.229044 | 0.997755 | -1.30208 | 0.0059739 | 0.142401 | -2.14675 | 1 |
| SGIP1 | 0.280842 | 0.986062 | 1.34128 | 0.171574 | 0.997755 | 1.57488 | 0.041893 | 0.263336 | 1.84707 | 1 |
| SIGMAR1 | 0.282466 | 0.986062 | -1.37327 | 0.423162 | 0.997755 | -1.2805 | 0.0192181 | 0.193771 | -2.08119 | 1 |
| VMO1 | 0.283148 | 0.986062 | 1.28195 | 0.335029 | 0.997755 | 1.28835 | 0.0168895 | 0.184923 | 1.87055 | 1 |
| RNASEK | 0.286087 | 0.986062 | -1.27463 | 0.263346 | 0.997755 | -1.29887 | 0.00504754 | 0.136683 | -2.28135 | 1 |
| ZNF283 | 0.288346 | 0.986062 | -1.28963 | 0.274268 | 0.997755 | -1.26011 | 0.0112867 | 0.163539 | -2.27192 | 1 |
| PHKG2 | 0.290137 | 0.986062 | -1.24747 | 0.437989 | 0.997755 | -1.19949 | 0.0203704 | 0.197755 | -1.88926 | 1 |
| TIMM13 | 0.293604 | 0.986062 | -1.37414 | 0.485151 | 0.997755 | -1.19675 | 0.00326708 | 0.127063 | -2.889 | 1 |
| PDPR | 0.294174 | 0.986062 | -1.14206 | 0.189184 | 0.997755 | -1.18266 | 0.00065351 | 0.083644 | -1.84938 | 1 |
| TRAPPC2L | 0.298666 | 0.986062 | -1.44826 | 0.448196 | 0.997755 | -1.27025 | 0.0188286 | 0.192067 | -2.40883 | 1 |
| LTBR | 0.298717 | 0.986062 | -1.31392 | 0.407849 | 0.997755 | -1.24259 | 0.00292018 | 0.120519 | -2.73223 | 1 |
| ARRB1 | 0.299434 | 0.986062 | -1.30882 | 0.363767 | 0.997755 | -1.21685 | 0.0207913 | 0.199816 | -2.01136 | 1 |
| WDR83OS | 0.30065 | 0.986062 | -1.23863 | 0.74965 | 0.997755 | -1.07763 | 0.00828328 | 0.150763 | -2.01217 | 1 |
| ERGIC3 | 0.305126 | 0.986062 | -1.2671 | 0.59961 | 0.997755 | -1.12477 | 0.0198717 | 0.196135 | -1.80833 | 1 |
| GBA | 0.309522 | 0.986062 | -1.41875 | 0.306187 | 0.997755 | -1.40646 | 0.0385783 | 0.255493 | -2.02893 | 1 |
| ANP32D | 0.312283 | 0.986062 | 1.29564 | 0.325168 | 0.997755 | 1.22329 | 0.00696993 | 0.143764 | 2.03311 | 1 |
| NR4A2 | 0.312339 | 0.986062 | 1.39119 | 0.156284 | 0.997755 | 1.65105 | 0.00526909 | 0.137855 | 3.53575 | 1 |
| RPS2P48 | 0.313261 | 0.986062 | -1.37568 | 0.50977 | 0.997755 | -1.24375 | 0.0119494 | 0.166263 | -2.37997 | 1 |
| NDUFA11 | 0.313349 | 0.986062 | -1.29832 | 0.503545 | 0.997755 | -1.23065 | 0.0115075 | 0.164355 | -2.53942 | 1 |
| RNASE2CP | 0.315531 | 0.986062 | 1.87619 | 0.495017 | 0.997755 | 1.3733 | 0.0436655 | 0.268381 | 3.15441 | 1 |
| RPSAP29 | 0.316376 | 0.986062 | -1.14619 | 0.231649 | 0.997755 | -1.18738 | 0.00126049 | 0.101767 | -1.84866 | 1 |
| CUTA | 0.316876 | 0.986062 | -1.19631 | 0.214188 | 0.997755 | -1.26237 | 0.00405614 | 0.130631 | -2.0014 | 1 |
| H2BC13 | 0.317127 | 0.986062 | -1.36117 | 0.343724 | 0.997755 | -1.3264 | 0.0183085 | 0.190207 | -2.34678 | 1 |
| DMAC2 | 0.323307 | 0.986062 | -1.3475 | 0.625439 | 0.997755 | -1.16019 | 0.0252528 | 0.215025 | -1.92515 | 1 |
| H2BC18 | 0.323562 | 0.986062 | -1.80889 | 0.442614 | 0.997755 | -1.61062 | 0.023979 | 0.210067 | -3.80201 | 1 |
| COX8A | 0.324662 | 0.986062 | -1.15197 | 0.547153 | 0.997755 | -1.08846 | 0.00222103 | 0.114633 | -1.81398 | 1 |
| MIR320A | 0.326826 | 0.986062 | 1.2787 | 0.140157 | 0.997755 | 1.55893 | 0.0106432 | 0.160263 | 2.25251 | 1 |
| USP36 | 0.328588 | 0.986062 | -1.16432 | 0.325729 | 0.997755 | -1.16291 | 0.00256006 | 0.114908 | -1.80795 | 1 |
| NHP2P1 | 0.332469 | 0.986062 | -1.24492 | 0.755508 | 0.997755 | -1.07228 | 0.0113188 | 0.163743 | -1.97335 | 1 |
| AFF4 | 0.334518 | 0.986062 | 1.25153 | 0.180804 | 0.997755 | 1.34796 | 0.0136184 | 0.173155 | 2.02152 | 1 |
| CUL9 | 0.335354 | 0.986062 | 1.25737 | 0.111643 | 0.997755 | 1.5435 | 0.0241063 | 0.21045 | 1.98989 | 1 |
| CLUL1 | 0.335357 | 0.986062 | 1.3321 | 0.367171 | 0.997755 | 1.28858 | 0.0229659 | 0.206093 | 1.93714 | 1 |
| IFIT5 | 0.335623 | 0.986062 | 1.65434 | 0.96786 | 0.997755 | -1.03521 | 0.0118258 | 0.166144 | 4.10232 | 1 |
| RFXANK | 0.336271 | 0.986062 | -1.215 | 0.360512 | 0.997755 | -1.22121 | 0.00167536 | 0.11118 | -2.53997 | 1 |
| ZNF17 | 0.337284 | 0.986062 | 1.2562 | 0.894341 | 0.997755 | 1.00477 | 0.0363032 | 0.24852 | 1.84172 | 1 |
| RAB5C | 0.33947 | 0.986062 | -1.31797 | 0.333971 | 0.997755 | -1.24085 | 0.0252627 | 0.215025 | -2.0533 | 1 |
| EMC8 | 0.339747 | 0.986062 | 1.36199 | 0.0889983 | 0.997755 | 1.82964 | 0.0165708 | 0.184156 | 2.30235 | 1 |
| TMEM259 | 0.341291 | 0.986062 | -1.18448 | 0.476915 | 0.997755 | -1.10016 | 0.00217972 | 0.114633 | -2.0523 | 1 |
| RPS15P4 | 0.342117 | 0.986062 | -1.24077 | 0.0502263 | 0.997755 | -1.66145 | 0.0144081 | 0.175417 | -1.85504 | 1 |
| VPS28 | 0.342574 | 0.986062 | -1.369 | 0.636536 | 0.997755 | -1.15017 | 0.00863054 | 0.150763 | -2.73273 | 1 |
| ZDHHC16 | 0.342723 | 0.986062 | -1.2388 | 0.587806 | 0.997755 | -1.13862 | 0.0167522 | 0.184578 | -1.80164 | 1 |
| PPAN-P2RY11 | 0.347219 | 0.986062 | -1.13312 | 0.0312871 | 0.997755 | -1.38201 | 0.00028123 | 0.0589926 | -2.10482 | 1 |
| MGAT5 | 0.348149 | 0.986062 | 1.43295 | 0.33896 | 0.997755 | 1.39799 | 0.0307176 | 0.235507 | 2.53463 | 1 |
| H2BC15 | 0.350527 | 0.986062 | -1.35734 | 0.353597 | 0.997755 | -1.3348 | 0.0290451 | 0.229939 | -2.22111 | 1 |
| MSTO1 | 0.350695 | 0.986062 | -1.27015 | 0.715374 | 0.997755 | -1.09713 | 0.0183224 | 0.190207 | -1.86356 | 1 |
| H2BC11 | 0.350792 | 0.986062 | -1.42806 | 0.139566 | 0.997755 | -1.64733 | 0.0390951 | 0.256389 | -2.3244 | 1 |
| RPL29 | 0.351964 | 0.986062 | -1.22191 | 0.170885 | 0.997755 | -1.34824 | 0.0124611 | 0.168752 | -1.89264 | 1 |
| SELPLG | 0.352845 | 0.986062 | -1.30566 | 0.0844584 | 0.997755 | -1.57121 | 0.0282908 | 0.22765 | -1.89198 | 1 |
| NT5DC2 | 0.353547 | 0.986062 | -1.17853 | 0.362568 | 0.997755 | -1.1666 | 0.00112036 | 0.0955974 | -2.27007 | 1 |
| CACNA1D | 0.354429 | 0.986062 | 1.21928 | 0.985849 | 0.999906 | -1.0204 | 0.00699809 | 0.143764 | 2.2165 | 1 |
| NDUFA13 | 0.355621 | 0.986062 | -1.2973 | 0.865807 | 0.997755 | -1.0515 | 0.00654781 | 0.143764 | -2.31905 | 1 |
| CCND3 | 0.361356 | 0.986062 | -1.3184 | 0.500518 | 0.997755 | -1.18171 | 0.00979651 | 0.156802 | -2.45824 | 1 |
| RPL18AP3 | 0.361995 | 0.986062 | -1.2162 | 0.357532 | 0.997755 | -1.23073 | 0.00935545 | 0.154253 | -1.98014 | 1 |
| IFITM3P1 | 0.367667 | 0.986062 | -1.4187 | 0.511886 | 0.997755 | -1.30255 | 0.00899723 | 0.15318 | -3.14055 | 1 |
| PLEKHM3 | 0.369931 | 0.986062 | 1.27162 | 0.439607 | 0.997755 | 1.27562 | 0.0351748 | 0.245398 | 1.96325 | 1 |
| ZNF805 | 0.371533 | 0.986062 | 1.20688 | 0.647159 | 0.997755 | -1.07298 | 0.00182679 | 0.111863 | 2.1886 | 1 |
| FADS2 | 0.371855 | 0.986062 | -1.23737 | 0.0829834 | 0.997755 | -1.4675 | 0.00443685 | 0.132323 | -2.10309 | 1 |
| CYC1 | 0.373714 | 0.986062 | -1.22757 | 0.509775 | 0.997755 | -1.13131 | 0.00990397 | 0.156822 | -2.10776 | 1 |
| HINFP | 0.375942 | 0.986062 | -1.30436 | 0.636669 | 0.997755 | -1.11439 | 0.0166402 | 0.184521 | -2.17957 | 1 |
| IFT43 | 0.378143 | 0.986062 | -1.27224 | 0.725147 | 0.997755 | -1.12073 | 0.0227866 | 0.205221 | -2.40116 | 1 |
| ALDOA | 0.380604 | 0.986062 | -1.19484 | 0.503628 | 0.997755 | -1.1486 | 0.00657632 | 0.143764 | -1.97346 | 1 |
| NLRC5 | 0.382879 | 0.986062 | 1.12635 | 0.0794284 | 0.997755 | 1.32842 | 0.00096004 | 0.0923534 | 1.9904 | 1 |
| ATP6V1F | 0.385429 | 0.986062 | -1.18014 | 0.644509 | 0.997755 | -1.0888 | 0.00861374 | 0.150763 | -1.88845 | 1 |
| NFXL1 | 0.385762 | 0.986062 | 1.26123 | 0.387829 | 0.997755 | 1.24282 | 0.0295402 | 0.2316 | 1.90597 | 1 |
| CORO1A | 0.387154 | 0.986062 | -1.27299 | 0.404466 | 0.997755 | -1.29425 | 0.00441173 | 0.132191 | -2.84876 | 1 |
| MCRS1 | 0.388942 | 0.986062 | -1.07913 | 0.554474 | 0.997755 | 1.04902 | 2.84E-05 | 0.0159042 | -2.01746 | 1 |
| USF3 | 0.396623 | 0.986062 | 1.22322 | 0.843412 | 0.997755 | 1.04694 | 0.0334534 | 0.241907 | 1.91442 | 1 |
| DDT | 0.400044 | 0.986062 | -1.16238 | 0.221495 | 0.997755 | -1.26544 | 0.00468139 | 0.136077 | -1.91639 | 1 |
| MPV17L2 | 0.40041 | 0.986062 | -1.26718 | 0.443782 | 0.997755 | -1.23651 | 0.0240998 | 0.21045 | -1.91393 | 1 |
| FAUP2 | 0.401257 | 0.986062 | -1.27205 | 0.691408 | 0.997755 | -1.12318 | 0.0175407 | 0.186561 | -2.11322 | 1 |
| SNORD26 | 0.402461 | 0.986062 | -1.42963 | 0.921002 | 0.997755 | 1.05233 | 0.0459806 | 0.272866 | -2.14306 | 1 |
| BFAR | 0.403737 | 0.986062 | -1.19182 | 0.970307 | 0.998003 | -1.00788 | 0.026684 | 0.220785 | -1.82076 | 1 |
| KLK5 | 0.404394 | 0.986062 | 1.17526 | 0.431304 | 0.997755 | -1.20776 | 0.0131256 | 0.171484 | 1.92886 | 1 |
| RPL12P14 | 0.409764 | 0.986062 | 1.29696 | 0.686083 | 0.997755 | -1.096 | 0.0494873 | 0.281679 | 1.93617 | 1 |
| TADA3 | 0.410388 | 0.986062 | -1.15938 | 0.929461 | 0.997755 | 1.02069 | 0.0174271 | 0.186338 | -1.81914 | 1 |
| CD81 | 0.412839 | 0.986062 | -1.23825 | 0.40034 | 0.997755 | -1.22436 | 0.014128 | 0.17487 | -1.97903 | 1 |
| MIR3936HG | 0.415241 | 0.986062 | -1.36679 | 0.273456 | 0.997755 | -1.7436 | 0.0151449 | 0.177609 | -3.68546 | 1 |
| SLC22A4 | 0.416376 | 0.986062 | -1.21603 | 0.100355 | 0.997755 | -1.60514 | 0.0172653 | 0.186309 | -2.11037 | 1 |
| ENTPD6 | 0.418278 | 0.986062 | 1.31963 | 0.160428 | 0.997755 | 1.57159 | 0.029564 | 0.2316 | 2.29765 | 1 |
| PBX2P1 | 0.422218 | 0.986062 | -1.26007 | 0.63118 | 0.997755 | -1.14845 | 0.00261309 | 0.115396 | -3.0102 | 1 |
| HOMEZ | 0.428124 | 0.986062 | 1.1904 | 0.301445 | 0.997755 | 1.24717 | 0.0235849 | 0.20791 | 1.89482 | 1 |
| DIPK1C | 0.431844 | 0.986062 | 1.28383 | 0.371708 | 0.997755 | 1.34813 | 0.00698388 | 0.143764 | 2.82912 | 1 |
| GSTP1 | 0.4325 | 0.986062 | -1.17088 | 0.487219 | 0.997755 | -1.15984 | 0.01003 | 0.157139 | -2.00834 | 1 |
| ZFP2 | 0.440743 | 0.986062 | -1.16716 | 0.559315 | 0.997755 | -1.09777 | 0.0013493 | 0.103851 | -2.58915 | 1 |
| MRTFA | 0.447245 | 0.986062 | -1.18406 | 0.425616 | 0.997755 | -1.17333 | 0.0112037 | 0.163083 | -2.03801 | 1 |
| CEBPA | 0.447369 | 0.986062 | -1.09395 | 0.190914 | 0.997755 | -1.18036 | 0.00041283 | 0.0686966 | -1.89995 | 1 |
| TMEM179 | 0.449546 | 0.986062 | 1.19774 | 0.257356 | 0.997755 | 1.36844 | 0.0173751 | 0.186338 | 1.98469 | 1 |
| TEX9 | 0.45322 | 0.986062 | 1.20019 | 0.286457 | 0.997755 | -1.23351 | 0.0280154 | 0.22634 | 1.81416 | 1 |
| SSR4 | 0.457268 | 0.986062 | -1.17433 | 0.273331 | 0.997755 | -1.31045 | 0.00460436 | 0.135555 | -2.33433 | 1 |
| FKBP7 | 0.457475 | 0.986062 | 1.12005 | 0.156434 | 0.997755 | 1.24117 | 0.00219999 | 0.114633 | 1.85467 | 1 |
| RPL34P26 | 0.458686 | 0.986062 | 1.51927 | 0.0471682 | 0.997755 | 2.65667 | 0.033793 | 0.243179 | 2.83447 | 2 |
| STAG3L5P | 0.712309 | 0.990452 | -1.13583 | 0.761715 | 0.997755 | -1.11046 | 0.045018 | 0.270556 | -1.95721 | 2 |
| WASHC3 | 0.463898 | 0.986062 | 1.21173 | 0.085279 | 0.997755 | 1.80057 | 0.0304871 | 0.234802 | 2.08171 | 1 |
| POLR2I | 0.467325 | 0.986062 | -1.33553 | 0.372529 | 0.997755 | -1.27164 | 0.0443074 | 0.268878 | -2.15581 | 1 |
| DCBLD2 | 0.468255 | 0.986062 | 1.21746 | 0.811903 | 0.997755 | 1.07274 | 0.0270673 | 0.222778 | 2.31414 | 1 |
| CRYGN | 0.47037 | 0.986062 | -1.23172 | 0.420432 | 0.997755 | -1.30774 | 0.0358109 | 0.246773 | -2.12069 | 1 |
| MYG1 | 0.475713 | 0.986062 | -1.15029 | 0.977463 | 0.998634 | -1.00686 | 0.0117367 | 0.165642 | -2.01014 | 1 |
| PMM1 | 0.477531 | 0.986062 | -1.21794 | 0.996272 | 0.999906 | 1.00523 | 0.0152552 | 0.177915 | -2.03855 | 1 |
| GLDC | 0.482068 | 0.986062 | 1.3331 | 0.727447 | 0.997755 | 1.11211 | 0.0399691 | 0.258856 | 1.96274 | 1 |
| HNRNPCP3 | 0.486073 | 0.986062 | -1.15617 | 0.863589 | 0.997755 | 1.04701 | 0.0213762 | 0.201353 | -1.82525 | 1 |
| SNORD43 | 0.49494 | 0.986062 | -1.14909 | 0.739455 | 0.997755 | -1.06988 | 0.00686769 | 0.143764 | -2.0468 | 1 |
| GALNTL5 | 0.500639 | 0.986062 | 1.16658 | 0.448281 | 0.997755 | 1.19074 | 0.0443972 | 0.268878 | 2.06341 | 1 |
| TESC | 0.506366 | 0.986062 | -1.12229 | 0.657617 | 0.997755 | -1.0586 | 0.00945677 | 0.154789 | -1.82782 | 1 |
| GDNF | 0.507505 | 0.986062 | -1.16005 | 0.269255 | 0.997755 | -1.42979 | 0.0449623 | 0.270556 | -1.90242 | 1 |
| FAM161A | 0.508775 | 0.986062 | 1.13774 | 0.758671 | 0.997755 | 1.07457 | 0.00641467 | 0.143764 | 2.03539 | 1 |
| APRT | 0.520388 | 0.986062 | -1.16744 | 0.43438 | 0.997755 | -1.17834 | 0.00880233 | 0.152168 | -1.95017 | 1 |
| DCXR | 0.520397 | 0.986062 | -1.16946 | 0.654257 | 0.997755 | -1.09463 | 0.00708837 | 0.144086 | -2.04042 | 1 |
| EIF5A2 | 0.522274 | 0.986062 | 1.1996 | 0.242455 | 0.997755 | 1.35899 | 0.0323529 | 0.238354 | 1.92625 | 1 |
| LGALS1 | 0.522618 | 0.986062 | -1.17302 | 0.549374 | 0.997755 | -1.1809 | 0.01574 | 0.180105 | -2.20065 | 1 |
| GPX1P2 | 0.527681 | 0.986062 | -1.12411 | 0.644219 | 0.997755 | -1.09735 | 0.0155402 | 0.179156 | -1.89202 | 1 |
| PKD1P1 | 0.529949 | 0.986062 | -1.13452 | 0.8401 | 0.997755 | -1.05264 | 0.00767268 | 0.148947 | -1.80346 | 1 |
| CXXC1 | 0.545519 | 0.986062 | -1.24801 | 0.760233 | 0.997755 | -1.09377 | 0.015207 | 0.177886 | -2.58743 | 1 |
| PLAUR | 0.546447 | 0.986062 | -1.1323 | 0.697639 | 0.997755 | -1.09283 | 0.0127123 | 0.169457 | -1.91769 | 1 |
| GRN | 0.548723 | 0.986062 | -1.14485 | 0.808175 | 0.997755 | -1.07022 | 0.00413446 | 0.130631 | -2.1668 | 1 |
| PTPRN | 0.548751 | 0.986062 | 1.19066 | 0.962663 | 0.997755 | 1.01291 | 0.0265388 | 0.220785 | 2.49623 | 1 |
| AMZ1 | 0.552656 | 0.986062 | 1.21403 | 0.952484 | 0.997755 | 1.10591 | 0.0450004 | 0.270556 | 1.89454 | 1 |
| PDE4DIP | 0.553848 | 0.986062 | 1.15007 | 0.286619 | 0.997755 | 1.29733 | 0.0275901 | 0.225002 | 1.81869 | 1 |
| TTC13 | 0.554204 | 0.986062 | 1.12824 | 0.0682012 | 0.997755 | 1.45978 | 0.00941214 | 0.154691 | 1.92833 | 1 |
| RNF5P1 | 0.554527 | 0.986062 | -1.25832 | 0.471447 | 0.997755 | -1.20566 | 0.0362479 | 0.24839 | -2.36608 | 1 |
| SNORD27 | 0.555725 | 0.986062 | -1.2057 | 0.846586 | 0.997755 | 1.07088 | 0.0464864 | 0.274174 | -1.80261 | 1 |
| ETFRF1 | 0.556075 | 0.986062 | 1.1778 | 0.083751 | 0.997755 | 1.52694 | 0.0012949 | 0.103475 | 2.70472 | 1 |
| TMEM161A | 0.56351 | 0.986062 | -1.23952 | 0.298976 | 0.997755 | -1.429 | 0.018997 | 0.193153 | -2.18687 | 1 |
| RABL2A | 0.566136 | 0.986062 | -1.19876 | 0.393714 | 0.997755 | -1.25071 | 0.0179746 | 0.188645 | -2.07619 | 1 |
| GPS2 | 0.566139 | 0.986062 | -1.09057 | 0.846287 | 0.997755 | 1.04522 | 0.00519846 | 0.136781 | -1.83753 | 1 |
| TRPV1 | 0.576442 | 0.986062 | -1.14354 | 0.355906 | 0.997755 | 1.25396 | 0.00475024 | 0.136077 | 2.25395 | 1 |
| TSPO | 0.580054 | 0.986062 | -1.21726 | 0.408174 | 0.997755 | -1.30285 | 0.0265654 | 0.220785 | -2.15502 | 1 |
| ORAI2 | 0.585721 | 0.986062 | -1.23717 | 0.765368 | 0.997755 | 1.00837 | 0.0435133 | 0.268165 | -2.01775 | 1 |
| RNF167 | 0.58613 | 0.986062 | -1.15944 | 0.336218 | 0.997755 | -1.27838 | 0.00808012 | 0.150763 | -2.21632 | 1 |
| TOR3A | 0.587129 | 0.986062 | 1.1063 | 0.588617 | 0.997755 | 1.10573 | 0.00551417 | 0.139102 | 1.87125 | 1 |
| WDR24 | 0.589541 | 0.986062 | 1.13461 | 0.654615 | 0.997755 | -1.0446 | 0.0115793 | 0.164814 | 1.95049 | 1 |
| C17orf50 | 0.591855 | 0.986062 | 1.22033 | 0.740123 | 0.997755 | 1.09035 | 0.0375506 | 0.252906 | 2.3015 | 1 |
| TEAD4 | 0.59741 | 0.987351 | -1.19914 | 0.375426 | 0.997755 | -1.29801 | 0.0236266 | 0.20791 | -2.07677 | 1 |
| BTNL8 | 0.605451 | 0.988937 | -1.13171 | 0.441911 | 0.997755 | -1.21688 | 0.0188339 | 0.192067 | -1.97645 | 1 |
| UNC80 | 0.606246 | 0.988937 | 1.23026 | 0.539048 | 0.997755 | 1.24519 | 0.00929127 | 0.154047 | 3.00088 | 1 |
| TMEM143 | 0.616244 | 0.989028 | 1.23787 | 0.816755 | 0.997755 | -1.11307 | 0.0284776 | 0.227994 | 2.96602 | 1 |
| ZNF136 | 0.623038 | 0.989699 | -1.07001 | 0.525011 | 0.997755 | 1.12727 | 0.0116123 | 0.164814 | 1.8 | 1 |
| LDOC1 | 0.626737 | 0.989714 | 1.12398 | 0.162123 | 0.997755 | -1.40728 | 0.0136623 | 0.173276 | 1.94823 | 1 |
| GATA4 | 0.627354 | 0.989714 | 1.12898 | 0.989386 | 0.999906 | 1.0381 | 0.0236199 | 0.20791 | 2.08291 | 1 |
| MTG1 | 0.647393 | 0.989744 | -1.15393 | 0.271622 | 0.997755 | -1.40457 | 0.0193962 | 0.194258 | -2.02528 | 1 |
| RCN3 | 0.655293 | 0.989744 | 1.32294 | 0.311398 | 0.997755 | 1.738 | 0.046133 | 0.273449 | 3.06092 | 1 |
| AMPD3 | 0.662364 | 0.989744 | 1.15586 | 0.187932 | 0.997755 | 1.56792 | 0.04488 | 0.270464 | 2.3233 | 1 |
| WBP1P2 | 0.66943 | 0.989744 | -1.16881 | 0.196404 | 0.997755 | -1.46038 | 0.04293 | 0.266792 | -2.18374 | 1 |
| C1QL4 | 0.672019 | 0.989744 | 1.13237 | 0.565512 | 0.997755 | -1.0509 | 0.0176641 | 0.187478 | 2.28866 | 1 |
| ATP6V0B | 0.673468 | 0.989744 | -1.08218 | 0.598011 | 0.997755 | -1.09601 | 0.00612797 | 0.142829 | -1.85479 | 1 |
| MIEF2 | 0.68046 | 0.989744 | 1.10067 | 0.388373 | 0.997755 | 1.36255 | 0.0223469 | 0.204622 | 2.43113 | 1 |
| GPATCH1 | 0.681159 | 0.989744 | 1.22479 | 0.319407 | 0.997755 | 1.49075 | 0.0406467 | 0.260453 | 2.36651 | 1 |
| CDHR5 | 0.693264 | 0.989856 | 1.08931 | 0.292935 | 0.997755 | 1.21904 | 0.008283 | 0.150763 | 1.80232 | 1 |
| EPM2A-DT | 0.695968 | 0.989856 | 1.15105 | 0.907819 | 0.997755 | -1.03573 | 0.040446 | 0.259736 | 1.84602 | 1 |
| HAVCR2 | 0.705318 | 0.989856 | 1.07076 | 0.113479 | 0.997755 | 1.37365 | 0.00747308 | 0.147115 | 1.98233 | 1 |
| SLC39A7 | 0.708241 | 0.989856 | 1.07051 | 0.914268 | 0.997755 | -1.02721 | 0.0159561 | 0.180513 | -1.87857 | 1 |
| WASH6P | 0.71695 | 0.990947 | -1.07164 | 0.4883 | 0.997755 | -1.14266 | 0.00879621 | 0.152168 | -1.84177 | 1 |
| ZNF275 | 0.71713 | 0.990947 | 1.12473 | 0.661189 | 0.997755 | 1.13099 | 0.0126259 | 0.168752 | 2.40531 | 1 |
| ISL2 | 0.77301 | 0.991808 | 1.16989 | 0.641221 | 0.997755 | 1.14032 | 0.0441408 | 0.268878 | 2.04501 | 1 |
| MGRN1 | 0.782352 | 0.991808 | -1.08726 | 0.468773 | 0.997755 | -1.12726 | 0.0361359 | 0.247848 | -1.96181 | 1 |
| C1orf21 | 0.788146 | 0.991808 | -1.05441 | 0.363754 | 0.997755 | 1.26113 | 0.013843 | 0.174483 | 2.12049 | 1 |
| GRAP | 0.788875 | 0.991808 | 1.14885 | 0.757952 | 0.997755 | -1.16533 | 0.0272938 | 0.223788 | -2.60994 | 1 |
| STAG3L1 | 0.794006 | 0.991808 | -1.09643 | 0.995618 | 0.999906 | 1.01303 | 0.0167525 | 0.184578 | -2.37973 | 1 |
| EYA2 | 0.807548 | 0.991808 | 1.05852 | 0.593745 | 0.997755 | -1.08202 | 0.00830217 | 0.150763 | 1.81115 | 1 |
| ARL3 | 0.814659 | 0.991808 | 1.11733 | 0.185217 | 0.997755 | 1.44394 | 0.0363304 | 0.24852 | 2.0168 | 1 |
| ARL6 | 0.820505 | 0.991808 | -1.04751 | 0.863416 | 0.997755 | 1.03367 | 0.0125853 | 0.168752 | -2.17696 | 1 |
| SNORD24 | 0.822061 | 0.991808 | -1.11294 | 0.436309 | 0.997755 | 1.21657 | 0.038788 | 0.256389 | -1.90297 | 1 |
| PRDM13 | 0.827127 | 0.991808 | 1.00832 | 0.90965 | 0.997755 | -1.04016 | 0.0200765 | 0.196765 | 2.1232 | 1 |
| DNAH3 | 0.835074 | 0.991808 | 1.08319 | 0.39632 | 0.997755 | 1.34366 | 0.0261059 | 0.21946 | 2.84505 | 1 |
| ZNF19 | 0.852001 | 0.991808 | 1.01253 | 0.0647053 | 0.997755 | 1.19591 | 1.61E-05 | 0.0105821 | 2.12613 | 1 |
| NSUN5P1 | 0.852946 | 0.991808 | 1.04927 | 0.920934 | 0.997755 | 1.06369 | 0.0109213 | 0.161078 | -2.31893 | 1 |
| KCTD19 | 0.866516 | 0.991865 | 1.07333 | 0.918756 | 0.997755 | 1.0032 | 0.0469283 | 0.274748 | 2.02997 | 1 |
| SNORD12C | 0.880088 | 0.991865 | 1.06303 | 0.511801 | 0.997755 | 1.29569 | 0.0342432 | 0.243758 | -2.15172 | 1 |
| MLXIP | 0.88016 | 0.991865 | -1.0173 | 0.116881 | 0.997755 | -1.38066 | 0.0214112 | 0.201353 | -1.84917 | 1 |
| TMPRSS2 | 0.88829 | 0.991865 | 1.03354 | 0.727758 | 0.997755 | 1.09614 | 0.0458601 | 0.272449 | 1.82867 | 1 |
| AMPD2 | 0.888484 | 0.991865 | -1.13405 | 0.63327 | 0.997755 | -1.22911 | 0.00858796 | 0.150763 | -2.54958 | 1 |
| LY6G5B | 0.903077 | 0.993835 | -1.05082 | 0.871493 | 0.997755 | -1.06257 | 0.0146619 | 0.176216 | -2.75389 | 1 |
| DLG4 | 0.907036 | 0.994994 | 1.04103 | 0.257199 | 0.997755 | 1.26878 | 0.0125882 | 0.168752 | 1.81193 | 1 |
| MPND | 0.913563 | 0.995421 | 1.00319 | 0.885962 | 0.997755 | 1.06194 | 0.019688 | 0.195624 | 2.58105 | 1 |
| FRMPD4 | 0.916155 | 0.995421 | 1.01236 | 0.846654 | 0.997755 | 1.06518 | 0.0457859 | 0.272246 | 1.80025 | 1 |
| PTOV1 | 0.918802 | 0.995798 | -1.018 | 0.618756 | 0.997755 | -1.09663 | 0.0163185 | 0.183257 | -1.81391 | 1 |
| AARSD1 | 0.921706 | 0.995798 | 1.00803 | 0.765566 | 0.997755 | 1.02983 | 0.00034839 | 0.0646482 | -1.98692 | 1 |
| GRIK2 | 0.926909 | 0.996823 | 1.05462 | 0.964465 | 0.997755 | -1.0083 | 0.0394059 | 0.256787 | 2.00222 | 1 |
| UBIAD1 | 0.938374 | 0.997796 | -1.02587 | 0.33186 | 0.997755 | -1.25637 | 0.0154047 | 0.178144 | -1.81618 | 1 |
| LAMTOR1 | 0.950315 | 0.997804 | 1.02855 | 0.980922 | 0.999496 | 1.02968 | 0.0075004 | 0.147115 | -2.01793 | 1 |
| PPIE | 0.961031 | 0.99817 | 1.00427 | 0.533337 | 0.997755 | -1.12107 | 0.00428426 | 0.130631 | -1.88907 | 1 |
| ZNF526 | 0.971042 | 0.998557 | 1.02668 | 0.983262 | 0.999906 | 1.07935 | 0.0331794 | 0.241382 | 2.09951 | 1 |
| KNDC1 | 0.972589 | 0.998557 | 1.18897 | 0.0279579 | 0.997755 | 4.49354 | 0.0396584 | 0.257451 | 3.29287 | 2 |
| ABTB1 | 0.975776 | 0.998557 | -1.0049 | 0.782285 | 0.997755 | 1.09705 | 0.0148313 | 0.176231 | 1.9321 | 1 |
| SYNGR2 | 0.983088 | 0.999795 | -1.00568 | 0.59079 | 0.997755 | -1.10424 | 0.01475 | 0.176231 | -1.97988 | 1 |
| FKRP | 0.00973917 | 0.986062 | 1.74173 | 0.00258449 | 0.997755 | 2.11405 | 0.0140813 | 0.17487 | 1.6668 | 1 |
| TTLL3 | 0.0181428 | 0.986062 | 1.89722 | 0.0231472 | 0.997755 | 1.84295 | 0.855661 | 0.945428 | 1.08332 | 2 |
| IGFLR1 | 0.0365455 | 0.986062 | 1.34271 | 0.00061175 | 0.577456 | 1.92186 | 0.325148 | 0.625649 | 1.13144 | 1 |
| CLEC4A | 0.0390731 | 0.986062 | 1.87607 | 0.0430211 | 0.997755 | 1.9478 | 0.114797 | 0.393237 | 1.62016 | 2 |
| LFNG | 0.0463427 | 0.986062 | 2.3559 | 0.0376218 | 0.997755 | 2.31996 | 0.0585109 | 0.300446 | 2.01196 | 2 |
| PID1 | 0.0623385 | 0.986062 | 1.40126 | 0.00409089 | 0.997755 | 1.88843 | 0.0180918 | 0.189356 | 1.56881 | 1 |
| LINC02610 | 0.0683473 | 0.986062 | -1.83884 | 0.028474 | 0.997755 | -1.97746 | 0.807202 | 0.925518 | 1.10555 | 1 |
| SLC16A5 | 0.0969745 | 0.986062 | -1.42621 | 0.0108737 | 0.997755 | -1.81608 | 0.417609 | 0.69759 | -1.13476 | 1 |
| RNF216P1 | 0.098873 | 0.986062 | -1.39918 | 0.0120447 | 0.997755 | -1.95731 | 0.0468519 | 0.274748 | -1.61651 | 1 |
| PAX3 | 0.151406 | 0.986062 | -1.50277 | 0.0416032 | 0.997755 | -1.85103 | 0.579256 | 0.804534 | -1.19039 | 1 |
| DMTN | 0.170187 | 0.986062 | 1.41893 | 0.017938 | 0.997755 | 1.89608 | 0.0206957 | 0.199549 | 1.7913 | 1 |
| PLCB3 | 0.189309 | 0.986062 | 1.44496 | 0.0403385 | 0.997755 | 1.97107 | 0.0610536 | 0.304824 | 1.74166 | 1 |
| SLC25A51 | 0.251212 | 0.986062 | -1.2772 | 0.0483035 | 0.997755 | -1.80223 | 0.242172 | 0.551413 | -1.37581 | 1 |
| NPAS1 | 0.38598 | 0.986062 | 1.3146 | 0.0468673 | 0.997755 | 2.06083 | 0.205838 | 0.510808 | 1.53168 | 1 |
| ZC3H10 | 0.396724 | 0.986062 | -1.42739 | 0.0339115 | 0.997755 | -2.24585 | 0.746899 | 0.896988 | 1.10943 | 1 |
| CALCA | 0.439169 | 0.986062 | 1.248 | 0.0357046 | 0.997755 | -1.81701 | 0.793257 | 0.919008 | 1.11823 | 1 |
| ISG15 | 0.588303 | 0.986062 | -1.35617 | 0.0492433 | 0.997755 | -2.37326 | 0.587471 | 0.808997 | 1.33026 | 1 |
| HFE | 0.889791 | 0.991865 | -1.00988 | 0.0279558 | 0.997755 | 2.0672 | 0.268495 | 0.575622 | 1.33131 | 1 |
| SH3BP5-AS1 | 0.00139656 | 0.405619 | 1.88566 | 0.0771551 | 0.997755 | 1.29461 | 0.173055 | 0.473445 | 1.21052 | 1 |
| CXorf65 | 0.00170246 | 0.467494 | 1.82392 | 0.0891163 | 0.997755 | 1.28091 | 0.567464 | 0.797596 | 1.07811 | 1 |
| TNFSF9 | 0.00315785 | 0.691203 | 2.30473 | 0.059985 | 0.997755 | 1.54239 | 0.0226516 | 0.205133 | 1.78722 | 1 |
| LIPF | 0.00396086 | 0.73853 | -2.43977 | 0.497668 | 0.997755 | 1.15265 | 0.188151 | 0.49087 | -1.36527 | 1 |
| LINC01905 | 0.00619586 | 0.891201 | 2.08127 | 0.357302 | 0.997755 | 1.20253 | 0.240841 | 0.549832 | 1.31422 | 1 |
| MAF | 0.0103821 | 0.986062 | 1.90457 | 0.481768 | 0.997755 | 1.14396 | 0.0375729 | 0.252906 | 1.61348 | 1 |
| MAGED4 | 0.0148176 | 0.986062 | 2.012 | 0.234602 | 0.997755 | 1.27191 | 0.160193 | 0.457803 | 1.36712 | 1 |
| BHLHE41 | 0.0157982 | 0.986062 | 1.82052 | 0.0932832 | 0.997755 | 1.43361 | 0.0295651 | 0.2316 | 1.66946 | 1 |
| TK2 | 0.0185596 | 0.986062 | 1.87678 | 0.312611 | 0.997755 | 1.2667 | 0.107451 | 0.382825 | 1.4424 | 1 |
| MCHR2-AS1 | 0.0192485 | 0.986062 | 3.00828 | 0.387102 | 0.997755 | 1.56836 | 0.349791 | 0.644639 | 1.39011 | 1 |
| ZNF672 | 0.0200362 | 0.986062 | 1.97079 | 0.591907 | 0.997755 | 1.13867 | 0.125088 | 0.410083 | 1.50393 | 1 |
| SPHK1 | 0.0212013 | 0.986062 | 2.03316 | 0.100939 | 0.997755 | 1.54405 | 0.196368 | 0.499116 | 1.37291 | 1 |
| RN7SL368P | 0.0259313 | 0.986062 | 2.39051 | 0.203535 | 0.997755 | 1.53799 | 0.525379 | 0.772505 | 1.37685 | 1 |
| KIAA0825 | 0.0277325 | 0.986062 | 1.96217 | 0.134834 | 0.997755 | 1.47255 | 0.146119 | 0.441192 | 1.44402 | 1 |
| SYT7 | 0.0297774 | 0.986062 | -2.2887 | 0.142602 | 0.997755 | -1.72216 | 0.929837 | 0.974484 | -1.00018 | 1 |
| ZNF726 | 0.0301941 | 0.986062 | 2.08475 | 0.38254 | 0.997755 | 1.39372 | 0.84475 | 0.941917 | 1.02821 | 1 |
| PARP14 | 0.0341682 | 0.986062 | 2.11453 | 0.478739 | 0.997755 | 1.21722 | 0.158317 | 0.45631 | 1.48818 | 1 |
| ERVK13-1 | 0.0396252 | 0.986062 | 1.85782 | 0.409829 | 0.997755 | 1.18052 | 0.14095 | 0.435737 | 1.45996 | 1 |
| KASH5 | 0.0421182 | 0.986062 | 8.18192 | 0.554312 | 0.997755 | 1.50105 | 0.857713 | 0.946149 | 1.13055 | 1 |
| LY6D | 0.0448566 | 0.986062 | 1.85335 | 0.0969496 | 0.997755 | 1.57759 | 0.126308 | 0.411482 | 1.4887 | 1 |
